# Ammonium Salts as Curing Agents to Obtain Ionic Epoxy Resins With a Thermoplastic‐to‐Thermoset Transition

**DOI:** 10.1002/adma.202516894

**Published:** 2025-11-22

**Authors:** Izabela Kurowska, Leire Unanue, Itxaso Calafel, Dorleta Otaegui, Jorge L. Olmedo‐Martínez, Lourdes Irusta, Daniel Vinagre, Nora Aranburu, Nerea Casado, Ibon Odriozola, David Mecerreyes

**Affiliations:** ^1^ POLYMAT University of the Basque Country UPV‐EHU Donostia‐San Sebastián 20018 Spain; ^2^ IKERBASQUE Basque Foundation for Science Bilbao 48009 Spain

**Keywords:** ammonium curing agents, curable prepregs, epoxy composites, epoxy thermosets, ionic networks, thermoplastic intermediates, two‐stage curing

## Abstract

A new class of epoxy thermosets is presented, prepared using ammonium salts as hardeners, enabling the direct formulation of networks with ionic functionality from simple, industrially available building blocks. The curing process occurs in two distinct thermal stages: the formation of a fusible thermoplastic‐like intermediate, followed by crosslinking into an infusible thermoset. This intermediate behavior is highly unusual in epoxy systems and allows the material to be processed using methods typically applied to thermoplastics, such as extrusion. The practical relevance of this system is demonstrated through the fabrication of enduring prepregs that can be shaped and handled as thermoplastics during processing, but form crosslinked thermosets upon curing. Finally, the cured matrix can be chemically solubilized in aqueous nitric acid under relatively mild conditions, allowing efficient recycling of intact carbon fibers. This work introduces a scalable platform for functional epoxy networks with broad processing and application potential.

## Graphical abstract

Ionic epoxy networks are prepared using ammonium salts as hardeners, leading to a two‐stage curing process with a thermoplastic‐like intermediate. This uncommon behavior enables extrusion and fabrication of thermoplastic prepregs that can be cured into thermoset composites. The resulting materials combine ionic functionality and versatile processing. They also enable chemical matrix dissolution for fiber recovery, while relying on simple, industrially available components.

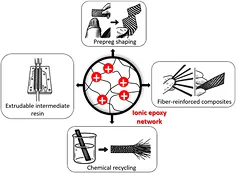

## 1 Introduction

Epoxy resins are among the most widely used thermosets in high‐performance materials, thanks to their outstanding mechanical, thermal, and chemical properties.^[^ ^1^, ^2^ ^]^ Their crosslinked networks, often formed through reactions between epoxides and amines, are well understood and broadly applied in sectors ranging from aerospace to electronics.^[^ ^3^ ^]^ Over time, however, the demands on epoxy systems have evolved, with increasing interest in endowing them with additional functionalities beyond their traditional roles.^[^ ^4^ ^]^

Commonly, two main strategies have been pursued to expand the processing versatility of epoxy systems. The first involves blending epoxy resins with thermoplastic polymers such as polyethersulfone (PES), polycaprolactone (PCL), or polyetherimide (PEI). These formulations display melt‐processable behavior before curing, enabling techniques like film extrusion or improved fiber impregnation. However, this strategy relies entirely on an external polymer phase to impart thermoplasticity and ultimately culminates in conventional crosslinking to form the final network.^[^ ^5^ ^]^ A second widely explored approach is the design of covalent adaptable networks (CANs),^[^ ^6^ ^]^ commonly referred to as vitrimers,^[^ ^7^ ^]^ which incorporate dynamic covalent bonds.^[^ ^8^, ^9^, ^10^ ^]^ These systems allow the network to rearrange under heat without passing through a true meltable state. While they offer reprocessability or self‐healing capabilities, their behavior fundamentally differs from classical thermoplastic‐to‐thermoset transitions.^[^ ^11^ ^]^

On the other hand, the integration of ionic functionalities into epoxy networks has gained interest for enabling new behaviors, such as ionic conductivity, enhanced interfacial adhesion, or responsiveness to electric fields, while preserving the structural benefits of thermosets.^[^ ^12^ ^]^ Networks bearing covalently bound ammonium groups are especially attractive, as they introduce permanent ionic character without relying on phase‐separated additives. This has been achieved either by synthesizing custom ionic monomers,^[^ ^13^, ^14^, ^15^ ^]^ or by adding quaternary ammonium additives to the formulation.^[^ ^16^ ^]^ While the former approach offers strong integration but requires multi‐step synthesis, the latter is more practical, yet the resulting ionic species are typically not covalently bound to the network, which may compromise long‐term stability. In some cases, tertiary amines and imidazole derivatives, commonly used as latent accelerators for epoxy curing, can react with epoxide rings to form covalently bound quaternary ammonium groups as part of the network.^[^ ^17^ ^]^ These species arise from nucleophilic ring opening of the epoxide, producing β‐hydroxyalkylammonium intermediates that remain chemically anchored within the crosslinked matrix. Although this mechanism introduces ionic character within the thermoset, the extent of functionalization is typically low, as these additives are used in catalytic amounts and compete with other reaction pathways. Moreover, the generation of ionic groups is incidental and not a primary design objective, limiting their effectiveness in applications requiring defined or high‐density ionic content.

**Figure 1:**
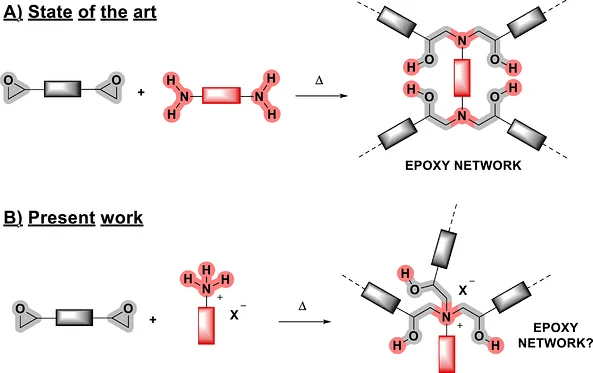
Conceptual comparison between: A) the conventional epoxy network formed by curing a diepoxide with a primary diamine, and B) the proposed use of ammonium salts as curing agents for epoxy resins.

In exploring simpler and easily scalable ways to design ionic epoxy networks, we were drawn to an idea that has long lingered in the background: the tertiary amines that naturally form during epoxy‐amine curing could, in principle, be quaternized to form ionic species. The idea is attractive in theory, but in practice, post‐curing modification of a rigid thermoset network proves highly impractical. This led us to consider a different question: what if the amine curing agent were already in its ammonium salt form? We reasoned that an ammonium ion (R─NH_3_⁺), bearing three labile N─H protons, might be able to engage in epoxide ring opening through each of these protons, potentially acting as a multifunctional hardener. This would contrast with the classical bifunctionality of neutral primary amines, where only two such reactions typically occur. In conventional systems, diamines (or polyamines) are required to build thermoset networks from bifunctional epoxy resins (**Figure** **1**A). While the reactivity of protonated amines is generally overlooked in epoxy chemistry, given that nucleophilic attack is commonly attributed to neutral or deprotonated amines, we were curious to examine how ammonium salts might behave in the presence of epoxides at different temperatures.

Thus, in this work we explore the use of ammonium salts as curing agents for epoxy resins (Figure 1B). Our approach is straightforward, avoids complex monomer synthesis, and introduces a new pathway to ionic thermosets using simple, industrially known building blocks.

## 2 Results and Discussion

### 2.1 Proof of Concept Using Discrete Model Compounds

To probe the reactivity of ammonium salts toward epoxides, a model reaction was carried out between three equivalents of butyl glycidyl ether (BGE) and one equivalent of the anilinium mesylate salt (**S1**), in bulk at 100 °C for 4 h. Aniline was selected as the primary amine due to its widespread industrial relevance, particularly in the production of 4,4′‐methylenedianiline (MDA), methylene diphenyl diisocyanate (MDI), and other key intermediates. Global aniline production exceeds six million tons annually, with a steady growth rate of ≈3–5% per year. Moreover, the world's first pilot plant for bio‐based aniline, developed by Covestro, was inaugurated in 2024 in Leverkusen, Germany, highlighting the growing interest in sustainable aromatic amines.

The mesylate anion was chosen for its low nucleophilicity, in contrast to anions such as chloride, which are known to react with epoxide rings and trigger undesired side reactions. Anilinium mesylate **S1** was easily prepared by adding methanesulfonic acid to a cold aniline solution in diethyl ether, then filtering the resulting salt. The high boiling point of methanesulfonic acid is also advantageous, as it reduces the risk of acid dissociation and evaporation during the curing process.

**Figure 2:**
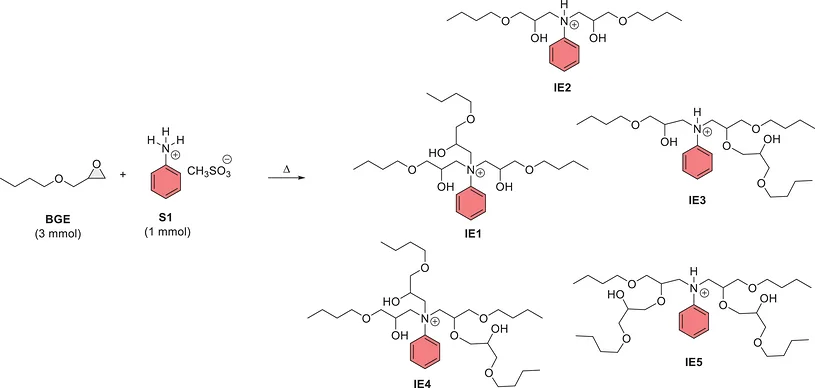
Reaction scheme illustrating the transformation of 3 mmol of butyl glycidyl ether (BGE) with 1 mmol of anilinium mesylate (**S1**), yielding a mixture of ionic adducts **IE1**–**IE5**.

Ultra performance liquid chromatography coupled to mass spectrometry (UPLC‐MS) analyses of the reaction mixture revealed a family of five adducts (**IE1**–**IE5**), corresponding to various degrees and modes of substitution on the ammonium center (**Figure** **2**). While **IE1** represents the ideal trisubstituted product formed by three consecutive N─H ring‐opening reactions, other species such as **IE3** and **IE5** suggest that secondary hydroxyl groups generated during the reaction can also act as nucleophiles, competing with remaining N─H sites. Such competing ring‐opening reactions by secondary alcohols are also observed in conventional epoxy‐amine systems, where β‐hydroxy groups formed in situ can further react with epoxides, leading to branched or network structures.^[^ ^18^ ^]^ Although precise quantification of each species could not be achieved, the UPLC–MS profile consistently shows the presence of all five adducts across multiple experiments, indicating a reproducible and complex reactivity pattern (Figure S1, Supporting Information).

Although this model reaction with BGE does not lead to network formation, it demonstrates at the molecular scale that ammonium salts bearing N─H bonds can undergo multiple epoxide ring‐opening events under thermal conditions. Notably, all adducts except the disubstituted **IE2** have already undergone at least three epoxide ring‐opening reactions, indicating the potential of **S1** to act as crosslinker when combined with diepoxides such as bisphenol A diglycidyl ether (DGEBA), as will be discussed in Section 2.2.

In this case, nuclear magnetic resonance (NMR) spectroscopy was not a viable tool for structural elucidation, due to the presence of a large number of stereoisomers and overlapping signals that severely complicate spectral interpretation. Instead, electrospray ionization mass spectrometry (ESI–MS) enabled discrimination between adducts sharing the same nominal mass. For instance, both **IE1** and **IE3** appear at m/z = 484.36, but only the protic species **IE3** exhibits an [M – 1 + 23]^+^ peak, consistent with deprotonation and Na⁺ adduction. The same reasoning applies to distinguish **IE4** from its protic analogue **IE5**. These results support the proposed structural assignments and confirm the presence of labile N─H groups in selected adducts (Figures S2–S6, Supporting Information).

### 2.2 Synthesis of Ionic Epoxy Networks

**Figure 3:**
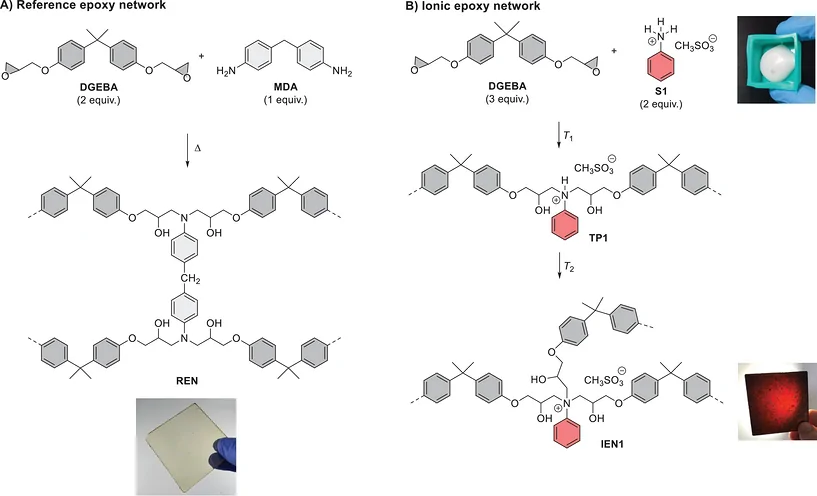
Synthetic scheme for the preparation of: A) reference epoxy network **REN**, and B) ionic epoxy network **IEN1**.

Once the concept was validated, we proceeded with the synthesis of epoxy networks. DGEBA was selected as the epoxy resin, given its widespread use as a standard monomer in industrial epoxy formulations. As a representative conventional epoxy formulation, two equivalents of DGEBA were reacted with one equivalent of MDA to produce the reference network **REN** (**Figure** **3**A).

For the preparation of the ionic epoxy network (**IEN1**), three equivalents of DGEBA were mixed with two equivalents of the solid hardener **S1**. The resulting paste was heated, and the reaction progress was monitored by Fourier‐transform infrared spectroscopy (FTIR). As the temperature approached 130–140 °C (Figure S7, Supporting Information), a marked shift in several absorption bands was observed. At this point, we expected the system to begin crosslinking. Instead, we found that the material had formed an amber colored, homogeneous, meltable intermediate, fully soluble in solvents such as THF and DMF. This was surprising, as such behavior is highly unusual for epoxy systems, where the curing process typically leads rapidly to an infusible thermoset. The intermediate, which we designate **TP1** (Figure 3B), shows a thermoplastic‐like behavior, suggesting a two‐stage curing process that could open the door to intriguing properties, as we will show later in this work. A preliminary in situ FTIR isotherm experiment carried out at 120 °C (monitoring the 916 cm^−1^ epoxy band) showed that the first stage proceeds with apparent first‐order kinetics (k = 2.1 × 10^−3^ s^−1^, R^2^ = 0.99) and reaches ≈60% conversion. This value is consistent with the stoichiometric ratio of the formulation, where two‐thirds of the epoxy groups are expected to react in this initial step, leading to the formation of the oligomeric, thermoplastic‐like intermediate (see Figure S8, Supporting Information).

Upon further heating to 170 °C, the system undergoes crosslinking to form an insoluble, reddish material, identified as the ionic epoxy network **IEN1** (Figure 3B). During this second transition, the following FTIR changes were still observed: (i) an increase in the O─H stretching band, indicating epoxy ring opening (Figure S9, Supporting Information); (ii) decrease of the absorption at 2648 cm^−1^, attributed to N^+^─H stretching (Figure S10, Supporting Information); (iii) a decrease in the N─H bending band at 1600 cm^−1^ (Figure S11, Supporting Information); (iv) an increase in absorbance in the 1320–1375 cm^−1^ region, associated with C─N bond formation (Figure S12, Supporting Information); (v) an increase in the C─O stretching band of secondary alcohols at 1120 cm^−1^ (Figure S13, Supporting Information); and (vi) a decrease in the characteristic epoxy ring bending band at 916 cm^−1^ (Figure S14, Supporting Information). The curing was completed by holding the sample at 170 °C for 2 h, resulting in the formation of a thermoset. Soxhlet extraction in THF for 48 h yielded gel contents of 98.8% for **REN** and 94.8% for **IEN1**, confirming the formation of highly crosslinked networks in both systems.

**Figure 4:**
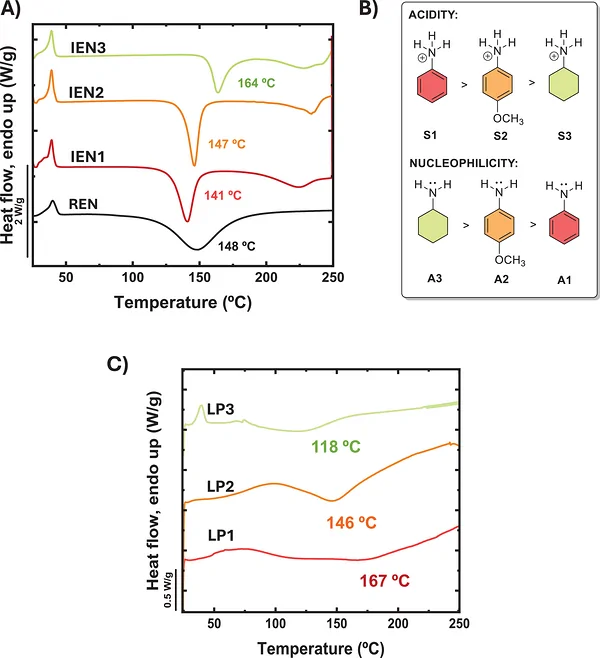
A) DSC heating scans (5 °C min^−1^) of the synthesized epoxy networks. B) Ammonium salts and their precursor amines used in this work, ordered by acidity and nucleophilicity, respectively. C) DSC plots of the reaction of DGEBA with amines **A1**‐**A3** to give **LP1**‐**LP3** for comparison.

This two‐stage curing behavior was further investigated by differential scanning calorimetry (DSC). When the initial mixture of DGEBA and **S1** was heated from room temperature to 250 °C at a rate of 5 °C min^−1^, two distinct exothermic peaks were observed (**Figure** **4**A, red curve). The first, relatively sharp peak appears at ≈141 °C and corresponds to the formation of the intermediate **TP1**. The second, broader peak at higher temperatures is attributed to the crosslinking step leading to the final network, **IEN1**. This behavior contrasts markedly with that of the reference epoxy network **REN**, which displays a single, much broader exothermic peak starting at lower temperatures (Figure 4A, black curve). In both cases, an endothermic peak below 50 °C is observed, corresponding to the melting of DGEBA. The molecular weight of the soluble intermediate **TP1** obtained after the first curing step was estimated by size exclusion chromatography (SEC), revealing a broad distribution with an average molecular weight of *M*_w_ = 4548 g mol^−1^. The dispersity (Đ = 2.24) indicates a moderately polydisperse system (Figure S15, Supporting Information).

At this stage, we extended our study to investigate the curing of DGEBA with different ammonium salts, focusing on how their molecular structure influences reactivity. To this end, mesylate salts **S2** and **S3** were prepared from their corresponding free amines: *p*‐anisidine (**A2**) and cyclohexylamine (**A3**), respectively (Figure 4B). The DSC curing profiles for all three systems are shown in Figure 4A, where the cured products are denoted as **IEN1**, **IEN2**, and **IEN3**, corresponding to DGEBA cured with **S1**, **S2**, and **S3**, respectively. When comparing the reactivity of the hardeners toward epoxy ring‐opening polymerization, the following trend was observed: aromatic (**S1**) > electron‐donating aromatic (**S2**) > aliphatic (**S3**).

At first glance, this trend appears counterintuitive, as the nucleophilicity of the corresponding free amines follows the opposite order (Figure 4B). Since oxirane ring‐opening typically proceeds via nucleophilic attack by the lone pair on the nitrogen, one might expect more nucleophilic amines to react faster. However, in our case, the amines are fully protonated (quaternized), and curing requires initial deprotonation of the ammonium group. Therefore, it seems that reactivity is governed primarily by the acidity of the salts rather than the nucleophilicity of the parent amines. Indeed, the more acidic salt **S1** (p*K*_a_H = 4.87)^[^ ^19^ ^]^ reacts more readily than **S2** (p*K*_a_H = 5.36),^[^ ^19^ ^]^ while **S3** (p*K*_a_H = 10.64)^[^ ^19^ ^]^ shows the lowest reactivity.

For comparison, the DSC thermograms of the free amines **A1**–**A3** reacting with equimolar amounts of DGEBA reveal a trend consistent with their nucleophilicity (Figure 4C). Notably, these curves exhibit broader exothermic peaks compared to those obtained with their corresponding salts (**S1**–**S3**), suggesting a less defined or slower reaction process.

**Figure 5:**
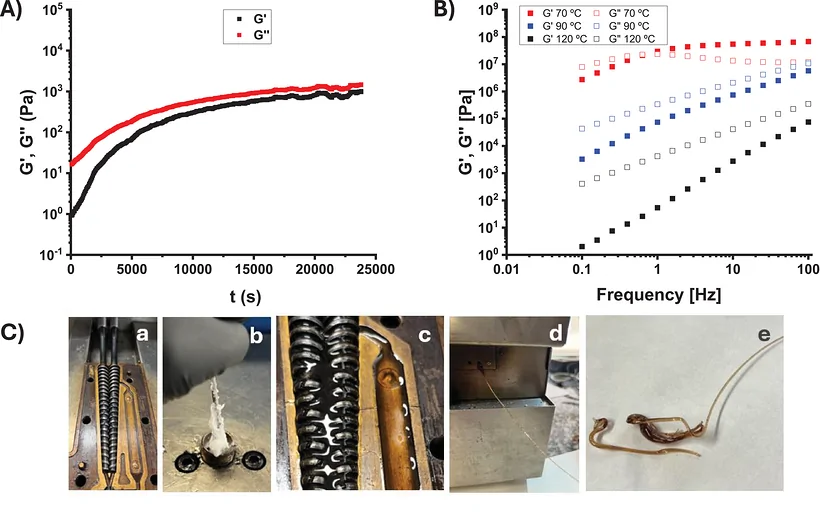
A) Evolution over time of the elastic (*G*') and viscous (*G*″) moduli of **TP1**, in a time sweep, at a constant frequency of 1 Hz and deformation in the linear viscoelastic region (LVR). B) Viscoelastic moduli (*G*′ and *G*″) of **TP1** at different temperatures (120, 90, and 70 °C), obtained from frequency sweep experiments performed within the linear viscoelastic regime. C) Extrusion of the intermediate **TP1** using a microextruder. (a) Twin‐screw system used for mixing and extrusion. (b) Feeding of the DGEBA and **S1** mixture into the extruder. (c) Visual inspection of the extruder after 30 min at 130 °C, showing homogeneous formation of **TP1**. (d) Extrusion of the melt through a 2 mm die. (e) Filament of **TP1** obtained after extrusion, confirming thermoplastic‐like behavior prior to curing.

To assess the thermoplastic‐like nature of the oligomeric species **TP1**, its rheological behavior was analyzed. A time sweep was first conducted at 150 °C to monitor the evolution of the storage modulus (*G*′) and loss modulus (*G*″) of a freshly mixed and homogenized DGEBA/**S1** formulation. As shown in **Figure** **5**A, both moduli increase steadily over time by ≈3 orders of magnitude, indicating the progressive formation of higher molecular weight species. After ≈400 min, both moduli reach a plateau, indicating that the oligomerization reaction has come to completion. At this stage, *G*″ remains above *G*′, indicating that the material retains predominantly viscous (liquid) behavior at 150 °C.

Next, frequency sweeps were performed at different temperatures to evaluate the dependence of the viscoelastic parameters on temperature at low frequencies. In thermosetting materials, even if they are partially cross‐linked or those that have significant restrictions in segmental mobility, the moduli typically show minimal sensitivity to temperature. Figure 5B shows the viscoelastic spectra obtained for **TP1** at 120, 90 and 70 °C. As observed, at 120 and 90 °C, the spectra correspond to the flow or terminal region of a typical linear polymer. In this region, the *G*″ modulus lies above *G*′ and exhibits a slope of –1, which is characteristic of thermoplastic polymers. As the temperature decreases (70 °C), both moduli increase, and a *G*′ = *G*″ crossover becomes discernible. This behavior indicates the appearance of the rubbery region in the spectrum and the proximity to the glass transition region, among other features.

To demonstrate the processability of **TP1**, a mixture of DGEBA and **S1** was introduced into a laboratory‐scale microextruder equipped with a twin‐screw system (Figure 5C, panel a). The formulation was mixed in a closed loop for 30 min at 130 °C, a temperature intentionally set below that used under static conditions, considering that the shear applied during extrusion would accelerate the reaction. After opening the extruder (panel c), a homogeneous melt was observed, confirming the in situ formation of **TP1**. The material was then extruded through a 2 mm die (panel d), producing continuous filaments (panel e) without signs of degradation or phase separation. This experiment confirms that the oligomeric intermediate **TP1** shows thermoplastic‐like behavior and can be processed by standard melt extrusion techniques prior to final curing.

Classical examples of processable epoxy–thermoplastic blends, as mentioned in the introduction, or epoxy‐derived thermoplastics include poly(hydroxy ether) architectures and early systems incorporating unsaturations that were later oxidatively crosslinked.^[^ ^20^ ^]^ However, these strategies differ substantially from the concept presented here. In our case, the thermoplastic character arises directly from the curing chemistry itself, without the need for added thermoplastic components, unsaturated sites, or dynamic exchange mechanisms. The process evolves from a homogeneous, melt‐processable intermediate into an ionic thermoset upon further heating. To the best of our knowledge, this two‐stage evolution represents a remarkable case within epoxy chemistry.

**Figure 6:**
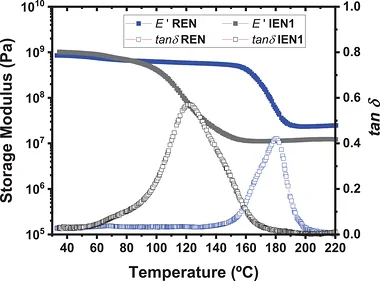
DMTA curves of **REN** and **IEN1**. The storage modulus (*E*′, left axis) and *tan δ* (right axis) are plotted as a function of temperature.

To assess the thermomechanical behavior of the thermoset materials, dynamic mechanical thermal analysis (DMTA) was performed. **Figure** **6** shows the DMTA spectra of **IEN1** and **REN**. The left Y‐axis corresponds to the storage modulus (*E*'), while the right Y‐axis represents the *tan δ* values. Both materials show similar stiffness at room temperature, with **IEN1** displaying a slightly higher modulus. However, the glass transition temperature (*T*_g_) of **IEN1**, identified as the maximum of the *tan δ* peak, is significantly lower (≈121 °C) than that of **REN** (≈180 °C).^[^ ^21^ ^]^ In addition, the broader *tan δ* peak of **IEN1** suggests increased structural heterogeneity or a less densely crosslinked network, consistent with its molecular architecture.

Thermogravimetric analysis (TGA) revealed that **IEN1**–**IEN3** exhibit slightly lower thermal stability compared to the reference network **REN** (Figure S16, Supporting Information), which may be attributed to the presence of ionic moieties or structural irregularities arising from the curing mechanism. Given the ionic character of **IEN1**, an increased affinity for polar solvents such as water could be expected. To evaluate the hydrophilicity of the final network, water uptake measurements were performed on cured **IEN1** samples. Circular specimens were immersed in deionized water at room temperature until saturation (48 h). After removing surface water, the samples were weighed, and the increase in mass relative to the dry weight corresponded to a water uptake of 11%. Under the same conditions, the reference epoxy network (**REN**), lacking ionic groups, showed only 1% water uptake. This substantial difference highlights the relatively strong affinity of **IEN1** for water, which can be attributed to the presence of ionic functionalities within the network. Nevertheless, this does not imply that the material absorbs significant moisture under ambient conditions. Indeed, thermogravimetric analysis of freshly cured and six‐month‐aged **IEN1** samples stored at 20–26 °C and 70–80% RH (Figure S17, Supporting Information) showed no significant weight loss below 150 °C, indicating negligible water uptake from the environment.

In line with this behavior, we hypothesised that the ability of **IEN1** to absorb water may also facilitate ionic transport through the network. The ionic conductivity of a water‐saturated **IEN1** sample was measured as a function of temperature, as shown in Figure S18 (Supporting Information). Although the absolute values of ionic conductivity are moderate (10^−^⁵ to 10^−^⁴ S cm^−1^), they are still remarkable for a rigid epoxy thermoset. These results demonstrate that ionic transport is possible within the network, likely enabled by the presence of fixed quaternary ammonium groups and their associated mobile counterions. In contrast, **REN** showed no measurable ionic conductivity under the same conditions. While a detailed investigation of ionic transport mechanisms lies beyond the scope of this study, the incorporation of fixed charges into the epoxy network may prove beneficial in various application areas, including antistatic coatings, structural energy storage and electroresponsive materials.

We next hypothesized that the thermoplastic‐like behavior of the intermediate **TP1** could offer unique advantages for the preparation of prepregs. Specifically, the impregnated sheets could be handled and shaped as thermoplastics prior to curing, and subsequently converted into a crosslinked thermoset. This behavior could enable several practical benefits, including: (i) no need for cold storage; (ii) non‐tacky surfaces, eliminating the need for protective liners; (iii) thermoformability, allowing bending and shaping with localized heat; and (iv) spot heating to temporarily induce tackiness, facilitating local fixation during component shaping.

**Figure 7:**
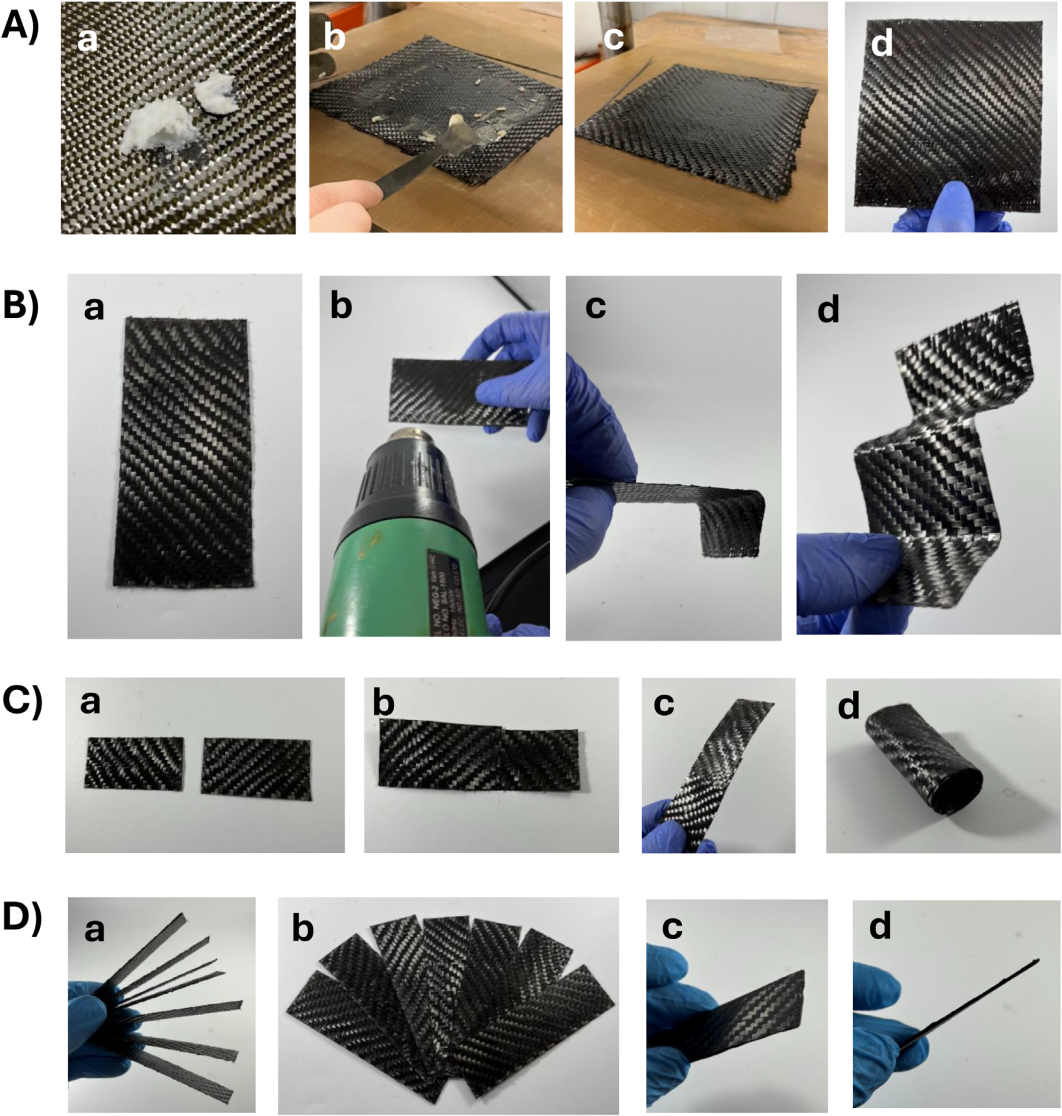
A) Preparation of enduring prepregs: A mixture of DGEBA and **S1** was applied over a CF sheet on a hot press at 150 °C (a). The solid mixture was manually spread across the surface in a manner similar to spreading butter on toast (b). Upon melting and partial reaction, the intermediate **TP1** formed in situ, homogeneously impregnating the CF sheet (c). After cooling, the prepreg was trimmed to the desired dimensions (d). B) Shaping the prepreg: A prepreg sheet (a) was locally heated with a heat gun until softening (b), reshaped, and retained its new form upon cooling (c,d). C) Joining of prepregs: Two prepreg pieces (a) were bonded by mild heating at the interface (b,c), creating a stable joint that allowed further manipulation (d). D) Preparation of CFRC: Seven prepreg sheets (a,b) were stacked and consolidated on a hot press at 160 °C for 30 min and 170 °C for 1 h, resulting in the final CFRC (c,d).

To validate these potential advantages, we prepared and manipulated carbon fiber (CF) prepregs using **TP1** as the resin. As shown in **Figure** **7**A, a mixture of DGEBA and **S1** was applied onto a CF sheet preheated on the lower plate of a hot press set to 150 °C. The system was held under these conditions for 1 h, during which the solid mixture melted and partially reacted, generating the thermoplastic‐like intermediate **TP1** that homogeneously impregnated the CF. Once cooled, the resulting prepreg could be easily handled and cut to shape. Figure 7B demonstrates the thermoformable nature of the material: localized heating with a heat gun softened the prepreg, enabling reshaping and fixation into stable forms upon cooling. Furthermore, as shown in Figure 7C, two prepreg sheets could be temporarily joined through the tackiness generated by mild heating. This reshaping and tackiness of the prepreg upon mild heating originate from the fact that **TP1** exhibits a glass transition temperature of ≈50 °C (see Figure S19, Supporting Information). Therefore, heating above this *T*_g_ softens the intermediate, enabling thermoplastic‐like manipulation prior to final curing. This reworkable processing window, enabled by the two‐stage curing mechanism, is uncommon in epoxy systems and could offer practical benefits in composite manufacturing. Previous developments based on vitrimer chemistry introduced the concept of *enduring prepregs*,^[^ ^22^ ^]^ which enabled storage at room temperature and also allowed reprocessing of the cured material itself, typically at temperatures well above its *T*_g_ (≈180–200 °C). In contrast, the present prepreg system offers comparable shelf stability, but the reshaping occurs at much lower temperatures (≈50–60 °C), prior to final crosslinking. Once cured, the composite behaves as a conventional epoxy thermoset and is not reprocessable. It is worth noting that the widespread use of epoxy thermosets in composites also stems from their processing advantages: they are directly shaped and cured from low‐viscosity liquid precursors, which greatly facilitates fiber impregnation and composite fabrication.^[^ ^23^ ^]^ In this respect, the distinctive advantage of the present two‐stage curing system is that, while retaining the benefits of conventional thermosets, it additionally provides a thermoplastic‐like processing window at relatively low temperatures prior to final crosslinking.

It should be noted that the poor solubility of the ammonium salt in epoxy resin at low temperature requires the impregnation step to be performed at ≈135 °C, where the system becomes sufficiently fluid to allow homogeneous mixing and fiber wetting. Although this condition restricts processing flexibility compared to conventional epoxy/amine formulations, the use of a finely micronized salt could improve dispersion and thus ease impregnation.

To demonstrate the practical applicability of the prepregs, a carbon fiber‐reinforced composite (CFRC) was fabricated by stacking multiple prepreg sheets and consolidating them via hot pressing. As shown in Figure 7D, seven layers of previously prepared prepregs were assembled and thermally cured at 160 °C for 30 min, followed by 170 °C for 1 h. Under these conditions, the **TP1** resin underwent crosslinking to yield **IEN1**, forming a compact and rigid composite. This process demonstrates the compatibility of the prepreg material with standard composite manufacturing techniques and confirms its ability to transition from a reshapeable intermediate into a fully cured structural material.

The mechanical properties of carbon fiber‐reinforced composites based on **REN** and **IEN1** were assessed by three‐point bending tests on 10‐ply laminates. Both materials exhibited high flexural strength, with values of 666 ± 35 MPa for **REN** and 680 ± 46 MPa for **IEN1**. The strain at break was also comparable (2.1 ± 0.2 % for **REN** and 2.2 ± 0.4 % for **IEN1**), while the flexural modulus of **IEN1** (41 ± 1.5 GPa) was slightly lower than that of **REN** (45 ± 1.3 GPa). These results show that the introduction of ionic functionalities in **IEN1** does not compromise mechanical performance, while enabling additional functionalities. Representative stress‐strain curves are provided in Figure S20 (Supporting Information). For comparison, the neat **IEN1** resin exhibited a flexural modulus of 2.8 ± 0.6 GPa, a flexural strength of 31.2 ± 6.1 MPa, and a strain at break of 1.3 ± 0.2 % (Figure S21, Supporting Information). Moreover, **TP1**‐based prepregs stored for six months under ambient laboratory conditions (20–26 °C, 70–80% RH) could still be hot‐pressed at 170 °C for 1 h to yield homogeneous, defect‐free composites, confirming that no premature curing occurred during storage.

**Figure 8:**
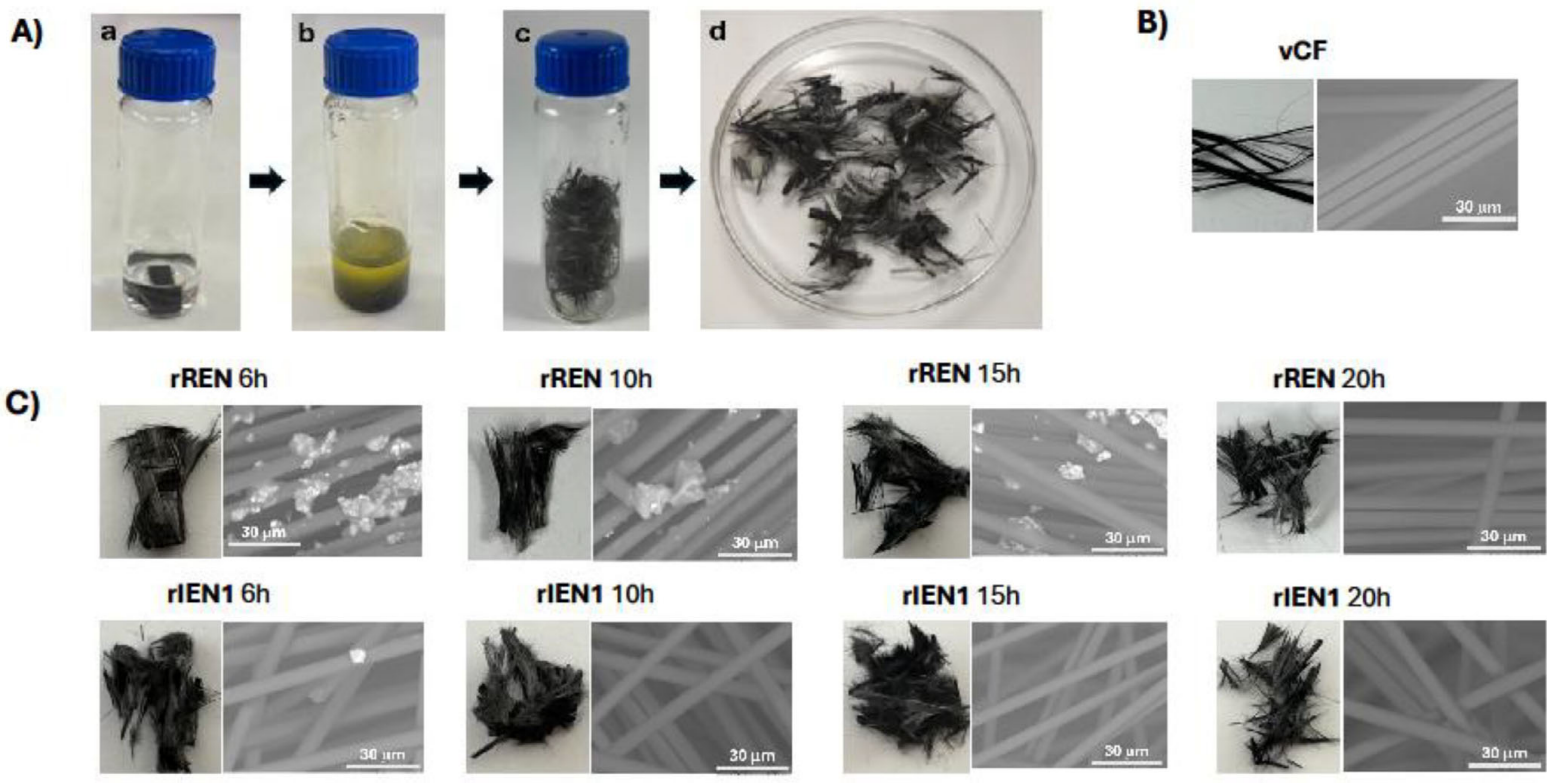
A) A small **IEN1** composite specimen immersed in aqueous HNO_3_ (a) was heated at 80 °C for 20 h, resulting in complete matrix dissolution (b). After subsequent washing with water and acetone (c), the released carbon fibers were isolated and dried (d). B) Photograph (left) and SEM micrograph (right) of virgin carbon fibers (**vCF**). C) Photographs (left) and SEM micrographs (right) of recycled carbon fibers from **REN**‐based composites (**rREN**, top panel) and **IEN1**‐based composites (**rIEN1**, bottom panel) at different nitric acid digestion times.

To further explore the potential of this system, we turned our attention to recyclability, an increasingly important consideration for fiber‐reinforced composites, where the carbon fiber component typically represents the highest material cost. Building on recent studies demonstrating that nitric acid can effectively decompose epoxy matrices under mild conditions while enabling the recovery of clean and reusable carbon fibers,^[^ ^24^, ^25^ ^]^ we employed this approach as a benchmark method. Composite specimens based on **IEN1** and on the non‐ionic reference **REN** were immersed in 8 m aqueous nitric acid at 80 °C under ambient pressure (**Figure** **8**A). A clear difference in degradation kinetics was observed: after 6 h, the **REN** composite showed only partial degradation, whereas the **IEN1** composite was almost completely dissolved, leaving nearly clean carbon fibers; after 10 h, the matrix of **IEN1** was fully removed while **REN** still retained visible residues; after 20 h, both systems were completely digested. Scanning electron microscopy (SEM micrographs of the recovered carbon fibers (Figure 8C) confirm complete removal of the resin from the fiber surface and preservation of the fiber morphology. Quantitative image analysis further revealed that the average fiber diameter remained essentially unchanged after recycling (6.85 ± 0.27 µm for **IEN1**‐derived fibers and 6.97 ± 0.11 µm for **REN**‐derived ones after 20 h of treatment, compared to 6.86 ± 0.14 µm for the virgin fibers), confirming that the oxidative treatment did not cause measurable erosion or damage to the carbon fibers. These results indicate that the ionic functionality in **IEN1** facilitates ingress of the aqueous oxidant and accelerates matrix removal compared to a non‐ionic epoxy under identical conditions.

## 3 Conclusion

We have demonstrated that ammonium salts can be used as curing agents to produce a new class of ionic epoxy thermosets. This curing approach, based on the direct reactivity of quaternized amines with oxirane groups, opens a previously unexplored direction in epoxy chemistry. By using simple and commercially available components, we have established a straightforward and scalable synthesis route that eliminates the need for complex monomer design. While the **IENs** described here were synthesized using pre‐formed ammonium salts, we note that an equivalent network can also be obtained via a one‐pot approach, by directly mixing the epoxy resin with the corresponding amine and acid as additive. This alternative route simplifies formulation and may be attractive for industrial applications.

A particularly distinctive feature of this system is its two‐stage curing behavior, which proceeds through a soluble, fusible oligomeric intermediate prior to thermoset network formation. This intermediate enables reshaping, thermoforming, and the generation of tackiness on demand, unlocking new opportunities for prepreg processing. The cured materials retain ionic functionality and can be chemically solubilized in nitric acid solution under mild conditions and at a faster rate than conventional non‐ionic epoxy networks, thereby enabling complete resin degradation and efficient recovery of intact carbon fibers.

Together, these characteristics position the system as a promising platform for next‐generation multifunctional composites. In particular, the combination of ionic character and carbon fiber compatibility suggests potential applications in structural energy storage devices, such as structural capacitors, where mechanical integrity and electrochemical performance must be integrated. Moreover, considering the commercial availability of biobased anilines and epoxy monomers such as diglycidyl ether of vanillyl alcohol (DGEVA), this strategy could be adapted to produce fully biobased ionic thermosets, using commercially available renewable epoxy and amine components.

## 4 Experimental Section

### Materials

Aniline (**A1**, TCI, 98%), *p*‐anisidine (**A2**, Sigma‐Aldrich, 99%) cyclohexylamine (**A3**, Acros Organics, 99%), methanesulfonic acid (Sigma‐Aldrich, 99%), bisphenol A diglycidyl ether (DGEBA, Sigma‐Aldrich, D3415), 4,4‘‐methylenedianiline (MDA, Sigma‐Aldrich, 99%), butyl glycidyl ether (BGE, Sigma‐Aldrich, 95%), 1,8‐diazabicyclo[5.4.0]undec‐7‐ene (DBU, Sigma‐Aldrich, 98%) and diethyl ether (Fisher, 99%) were used as received, without further purification. Carbon‐fiber fabrics (3K 2 × 2 twill weave, 200 g/m^2^, AS4C, Hexcel) were supplied by Castro Composites.

### Synthesis of Ammonium Mesylate Salts **S1**–**S3**

The corresponding amine (**A1**–**A3**, 0.2 mol) was dissolved in 450 mL of diethyl ether and cooled in an ice bath. Methanesulfonic acid (0.2 mol) was then added dropwise under stirring. The resulting white precipitate was filtered, washed with diethyl ether (3 × 200 mL) and analyzed by NMR. All the spectra were consistent with the expected structures. Anilinium mesylate (**S1**): ^1^H NMR (300 MHz, DMSO‐d_6_) δ 9.87 (s, 3H, NH_3_^+^), 7.55–7.45 (m, 2H, Ar─H), 7.45–7.34 (m, 3H, Ar─H), 2.42 (s, 3H, CH_3_SO_3_^−^).^13^C NMR (75 MHz, DMSO‐d_6_) δ 131.8, 129.6, 127.8, 122.8, 39.5. *p*‐Anisidinium mesylate (**S2**): ^1^H NMR (300 MHz, DMSO‐d_6_) δ 9.92 (s, 3H, NH_3_^+^), 7.38–7.25 (m, 2H, Ar─H), 7.13–6.98 (m, 2H, Ar─H), 3.76 (s, 3H, OCH_3_), 2.43 (s, 3H, CH_3_SO_3_^−^).^13^C NMR (75 MHz, DMSO‐d_6_) δ 158.6, 124.2, 123.8, 114.7, 55.2, 39.5. Cyclohexylammonium mesylate (**S3**): ^1^H NMR (300 MHz, DMSO‐d_6_) δ 7.80 (s, 3H, NH_3_^+^), 2.93 (m, 1H, ─CH─), 2.40 (s, 3H, CH_3_SO_3_^−^), 2.02–1.52 (m, 5H, ─CH_2_─), 1.38–0.91 (m, 5H, ─CH_2_─). ^13^C NMR (75 MHz, DMSO‐d_6_) δ 49.0, 39.5, 30.0, 24.3, 23.5.

### Model Reaction Between BGE and **S1**

BGE (3 mmol, 0.39 g) and ammonium mesylate salt **S1** (1 mmol, 0.19 g) were mixed in a round‐bottom flask and heated in bulk at 100 °C for 4 h under magnetic stirring. After cooling to room temperature, the crude mixture was analyzed without further purification. The formation of **IE1** was confirmed by UPLC‐MS (theoretical m/z = 484.3638, experimental m/z = 484.3633). Other products such as **IE2**, **IE3**, **IE4** and **IE5** were also identified (see Figure 2 and Supporting Information). ^1^H NMR analysis was not suitable due to extensive signal overlap caused by multiple stereoisomers. However, ESI‐MS allowed discrimination between protic and non‐protic species based on the presence or absence of sodium adduct peaks (see Figures S2–S6, Supporting Information).

### Preparation of **IEN1**‐**IEN3**

The general procedure for preparing ionic epoxy networks **IEN1**–**IEN3** consisted of mixing DGEBA with the corresponding ammonium salt in a 3:2 molar ratio, followed by thermal treatment in molds. DGEBA (3 mmol, 1.02 g) and **S1** (2 mmol, 0.38 g) were mixed in a mortar to form a homogeneous paste. The mixture was then transferred to a mold and heated to 150 °C for 30 min. During this step, a viscous intermediate (**TP1**) was formed. Upon further heating to 160 °C for 30 min and 170 °C for 1 h, the material underwent thermal crosslinking to yield the final ionic epoxy network (**IEN1**), which appeared as a rigid, reddish thermoset. The curing process was monitored by FTIR spectroscopy, showing the progressive consumption of epoxide groups and the appearance of characteristic bands consistent with O─H and C─N bond formation. Notably, the O─H stretching band increased, the epoxy bending at 916 cm^−1^ decreased, and the signal at 2648 cm^−1^ (associated with N─H⁺ stretching) disappeared. The intermediate **TP1** was soluble in THF and DMF, while the final **IEN1** was insoluble, as expected for a crosslinked thermoset. **IEN2** was prepared analogously, using **S2** as the hardener. For **IEN3**, based on **S3**, DBU was added as a catalyst at 3 mol% relative to the salt, to compensate for the lower reactivity of the system.

### Preparation of **REN**

DGEBA (4 mmol, 1.36 g) and MDA (2 mmol, 0.40 g) were placed in a round‐bottom flask and stirred under vacuum at 80 °C for 1 h to ensure complete homogenization. The resulting liquid was then transferred into a mold and cured sequentially at 150 °C for 30 min, 160 °C for 30 min, and finally at 180 °C for 1 h. Upon cooling, **REN** was obtained as a rigid, colorless thermoset.

### Preparation of Carbon Fiber‐Based Prepregs Using Intermediate **TP1**

Prepreg sheets were prepared from **TP1**, composed of a mixture of DGEBA and **S1** in a 3:2 molar ratio. The solid components were first ground in a mortar to obtain a homogeneous paste. A square of carbon fiber fabric was preheated on the lower plate of a hot press set to 150 °C and protected by a teflon film. The resin paste was then applied onto the warm fabric surface using a spatula, spreading it evenly in a manner similar to buttering toast. After 30 min under these conditions, the resin melted and reacted to give **TP1**, forming a continuous and uniform layer. After cooling to room temperature, the borders were trimmed to obtain prepreg sheets with final dimensions of 12 × 12 cm.

### Fabrication of CFRCs from **IEN1**

Seven to ten prepreg sheets made from **IEN1** (10 × 2.5 cm) were stacked between Teflon films and placed in a hot press. The stack was cured at 170 °C for 2 h under a pressure of 10 bar. During this step, the intermediate (**TP1**) underwent complete crosslinking, yielding a compact and rigid carbon fiber‐reinforced composite based on the ionic epoxy network (**IEN1**). For the three‐point bending tests, ten **IEN1** prepreg plies (15 × 15 cm) were stacked and cured under the same conditions.

### Fabrication of CFRCs from **REN**

For the preparation of the REN‐based composite used in three‐point bending tests, ten layers of carbon fiber fabric (15 × 15 cm) were individually impregnated with a liquid mixture of DGEBA and MDA and then stacked. The laminate was placed between Teflon sheets, introduced into a hot press, and cured using the following thermal profile: 150 °C for 30 min, 160 °C for 30 min, and 180 °C for 1 h.

### Chemical Recycling Procedure

CFRC specimens based on **IEN1** or **REN** were immersed in aqueous nitric acid (8 M HNO_3_, 16 mL per gram of sample) at 80 °C for 20 h. After this period, complete matrix degradation was observed. The released carbon fibers were collected, thoroughly rinsed with deionized water and acetone, and dried at 80 °C.

### Methods

**Fourier transform infrared (FTIR)** spectra were recorded using a Thermo Nicolet 6700 spectrometer in transmission mode equipped with a Specac 2093 heating cell. Data were collected by averaging 16 scans at a resolution of 4 cm^−1^. For kinetic measurements, isothermal FTIR experiments were performed at 120 °C, acquiring one spectrum every 22 s. The conversion of epoxy groups was determined from the decrease of the characteristic absorption band at 916 cm^−1^ as a function of time. The normalized absorbance of this band served as the basis for calculating the conversion, as defined by the following equation:

(1) $$p=1-\frac{A_{916(t)}}{A_{916(t0)}}$$

where A_916(t)_ and A_916(t0)_ represent the integrated areas of the epoxy ring absorption band at time t and at the initial time t_0_, respectively. **Ultra Performance Liquid Chromatography‐Mass Spectrometry (UPLC‐MS)**: Chromatographic separation was performed on an ACQUITY UPLC FTN system (Waters, Milford, MA, USA) equipped with an ACQUITY UPLC BEH C18 column (2.1 × 50 mm, 1.7 µm). The column temperature was maintained at 40 °C, and the sample manager at 10 °C. The mobile phases consisted of water with 0.1% formic acid (solvent A) and acetonitrile with 0.1% formic acid (solvent B). A linear gradient was applied from 5% to 95% B over 5 min, followed by re‐equilibration to initial conditions. The flow rate was 0.4 mL min^−1^, and the injection volume was 5 µL. Mass spectrometric detection was carried out on a Synapt XS high‐resolution mass spectrometer (Waters, Wilmslow, UK) equipped with an electrospray ionization (ESI) source operating in positive ion resolution mode. Data were acquired over the m/z range 100–3500. The source parameters were as follows: capillary voltage 3.2–3.4 kV, sampling cone 30 V, source offset 30 V, source temperature 130 °C, and desolvation temperature 500 °C. Nitrogen was used as desolvation gas (800 L h^−1^) and cone gas (50 L h^−1^). The nebulizer gas pressure was set to 5.9 bar. The scan time was 1.0 s. Data acquisition and processing were performed using MassLynx 4.2 software (Waters, Wilmslow, UK). **Differential scanning calorimetry (DSC)** analyses were conducted on a DSC25 instrument (TA Instruments) equipped with an intracooler, under a nitrogen atmosphere. ≈5 mg of sample was weighed and sealed in aluminum pans. Calibration was performed using pure indium and tin standards. Samples were typically heated to 250 °C, cooled to 0 °C, and reheated to 250 °C, all at a rate of 5 °C min^−1^. Data were analyzed using TRIOS software. **Thermogravimetric analysis (TGA)** was carried out on a PerkinElmer TGA 8000 instrument under a nitrogen atmosphere. Samples of ≈10 mg were heated from 40 to 800 °C at a rate of 10 °C min^−1^. Weight loss (%) as a function of temperature was analyzed using Pyris software. **Size exclusion chromatography (SEC)** analyses were performed in CHCl_3_ using a PL‐GPC 50 system (Agilent Technologies) equipped with an integrated refractive index detector. The setup included a guard column (PLgel, 5 µm, 50 × 7.5 mm) and two PLgel Mixed‐C columns (5 µm, 300 × 7.5 mm) connected in series (Agilent). Measurements were conducted at 40 °C with a flow rate of 1 mL min^−1^. Molar mass averages and distributions were calculated using Agilent GPC software and a calibration curve based on polystyrene standards (*Mp* = 162–3,187,000 g mol^−1^). **The gel content** of the cured resins was determined by Soxhlet extraction in THF for 48 h. Samples (≈200 mg) were placed in cellulose thimbles and extracted under reflux. After extraction, the insoluble fraction was dried under vacuum at 80 °C until constant weight. The gel content was calculated according to the Equation:

(2) $$gel\;content\left(\%\right)=\frac{m_{2}}{m_{1}}\times 100\%$$

where m_1_ is the initial dry weight of the sample and m_2_ is the dry weight of the insoluble material after extraction. **Viscoelastic properties** were measured using a Physica MCR 301 torsional rheometer (Anton Paar, Austria) equipped with parallel plate geometries of 50, 20, or 8 mm in diameter, depending on the sample dimensions. Time sweep experiments were performed at 150 °C, at a fixed frequency of 1.0 Hz and within the LVR, previously determined by strain sweep tests at the same temperature. Frequency sweep tests were conducted in the range of 0.1 to 100 Hz at 70, 90, and 120 °C, applying a constant deformation within the LVR. **Dynamic mechanical thermal analysis (DMTA)** was performed to assess the mechanical properties and glass transition temperatures of the materials. Measurements were carried out in tension mode using a Triton 2000 DMA instrument (Triton Technology, Mansfield, MA, USA). Rectangular specimens were heated from 20 °C to 220 °C at a constant rate of 4 °C min^−1^, under a fixed frequency of 1.0 Hz and a displacement amplitude of 15 µm. The glass transition temperature (*T*_g_) was determined as the temperature corresponding to the maximum of the tan δ curve. **Electrochemical impedance spectroscopy (EIS)** was performed using an Autolab 302 N potentiostat/galvanostat at various temperatures. Prior to each measurement, samples were equilibrated for 20 min. Specimens were placed between two stainless steel electrodes (surface area: 0.5 cm^2^), and impedance spectra were recorded from 100 kHz to 1 Hz using a 10 mV amplitude. Sample thickness ranged from 0.8 to 0.1 mm. Before each conductivity measurement, samples were immersed in water for 48 h. **Three‐point bending** tests were performed in accordance with ASTM D790 (Type I, Procedure A) using an Instron 5569 universal testing machine. A crosshead speed of 0.85 mm min^−1^ and a support span length of 32 mm were employed. Bending specimens measuring 2 x 12.7 x 50.8 mm^3^ were cut from the rectangular composite sheets obtained as described previously. The flexural elasticity modulus (*E_b_*), strength (σ_*fM*_), and strain at break (ε_*fB*_) were determined from the resulting stress‐strain curves. **Scanning Electron Microscopy (SEM)** micrographs were taken with an Analytical TableTop Microscope/Benchtop SEM TM3030 (Hitachi, Japan). EDS detector was used with a 15 kV beam accelerating voltage and the software used was Quantax 70. **Quantitative analysis of the fiber diameter** was performed on SEM micrographs using ImageJ software (NIH, USA). For each sample, ≈20 fibers were measured to determine the average diameter and standard deviation.

## Conflict of Interest

The authors declare no conflict of interest.

## Supporting information



Supporting Information

## Data Availability

The data that support the findings of this study are available in the supplementary material of this article.
