# Supplementary material for: Ammonium Salts as Curing Agents to Obtain Ionic Epoxy Resins With a Thermoplastic‐to‐Thermoset Transition
Source: Adv Mater. 2025 Nov 22;38(51):e16894. doi: 10.1002/adma.202516894 (PMC13569076; doi:10.1002/adma.202516894)
Supplement: Supplementary file 1 [file ADMA-38-e16894-s001.pdf]

Supporting Information

Ammonium Salts as Curing Agents to Obtain Ionic Epoxy Resins with a Thermoplastic-to-Thermoset Transition

Izabela Kurowska, Leire Unanue, Itxaso Calafel, Dorleta Otaegui, Jorge L. Olmedo-Martínez, Lourdes Irusta, Daniel Vinagre, Nora Aranburu, Nerea Casado, Ibon Odriozola, and David Mecerreyes**

Supplementary Figures

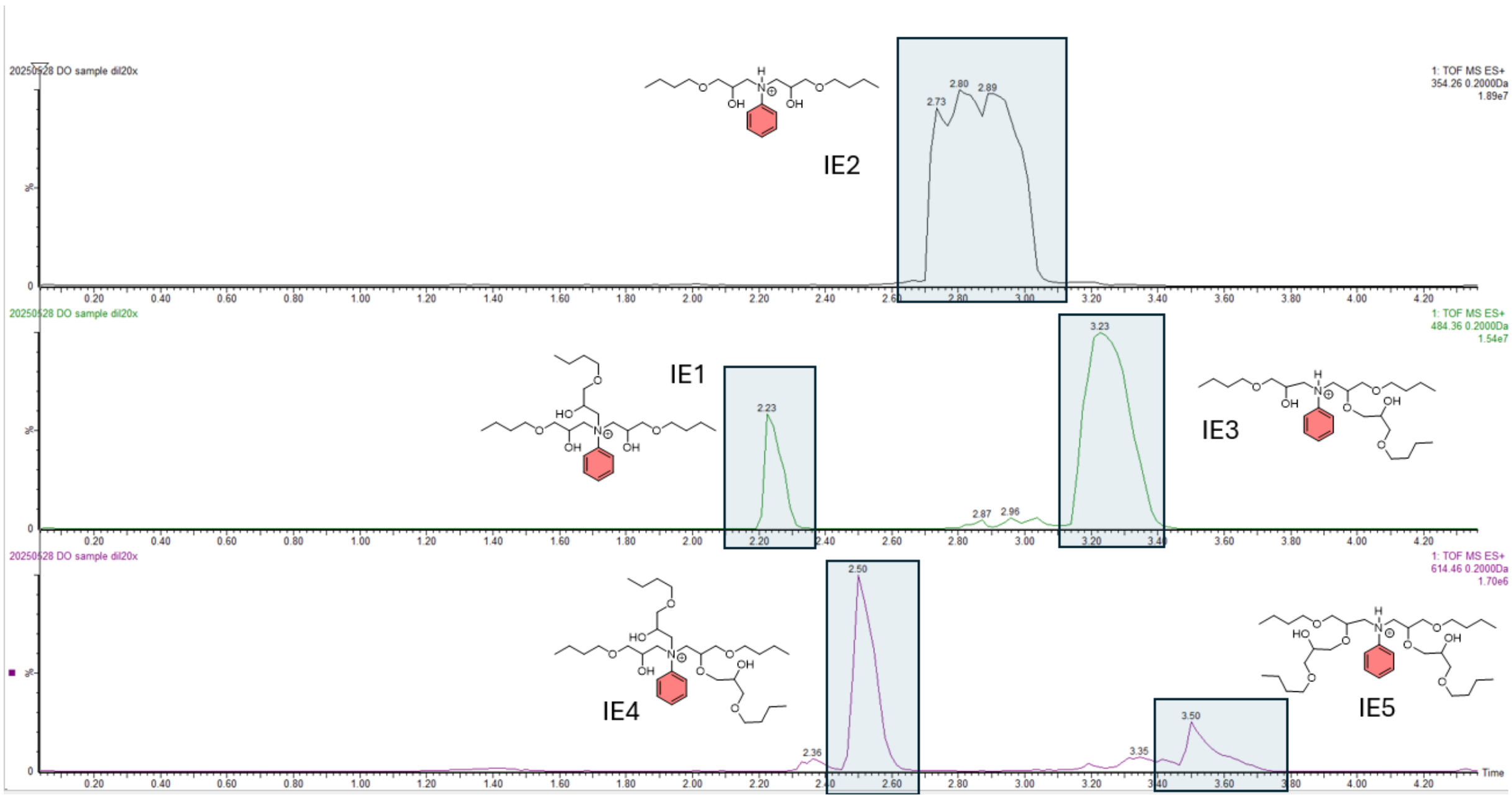

Figure S1. Analytical HPLC-MS chromatograms showing the individual peaks corresponding to the **IE1**–**IE5** adducts formed upon reaction of BGE (3 equiv) with **S1** (1 equiv).

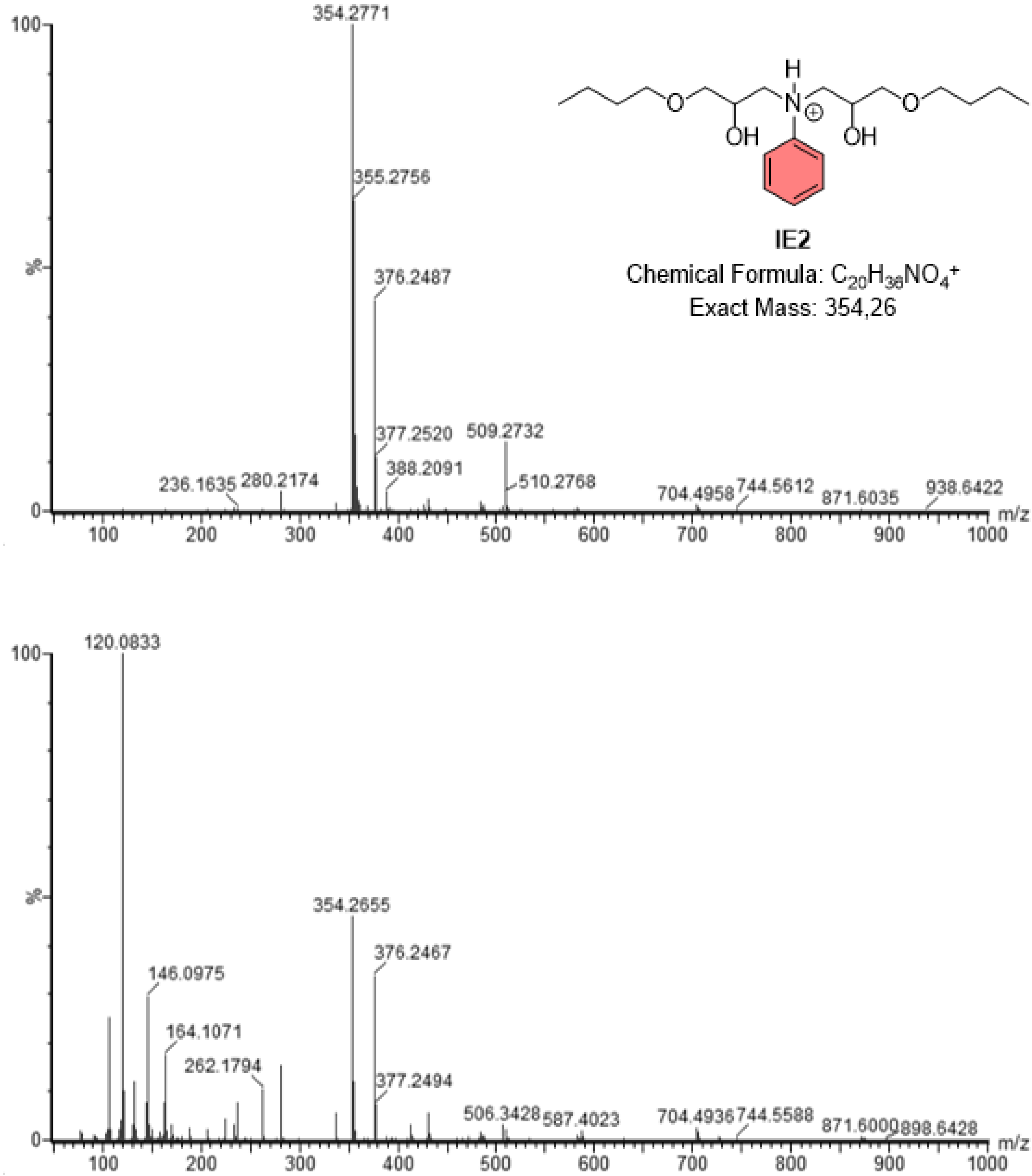

Figure S2. Electrospray ionization mass spectrum (ESI-MS, top) and tandem mass spectrum (MS/MS, bottom) of adduct **IE2** recorded in positive mode. The parent ion at *m/z* = 354.27 corresponds to the [M^+] species with formula $C_{20}H_{36}NO_4^+$, consistent with the expected structure of **IE2**. A secondary peak at *m/z* = 376.25 is also observed, corresponding to the sodium adduct $[M - H + Na]^+$. The MS/MS spectrum confirms the assignment through characteristic fragment ions.

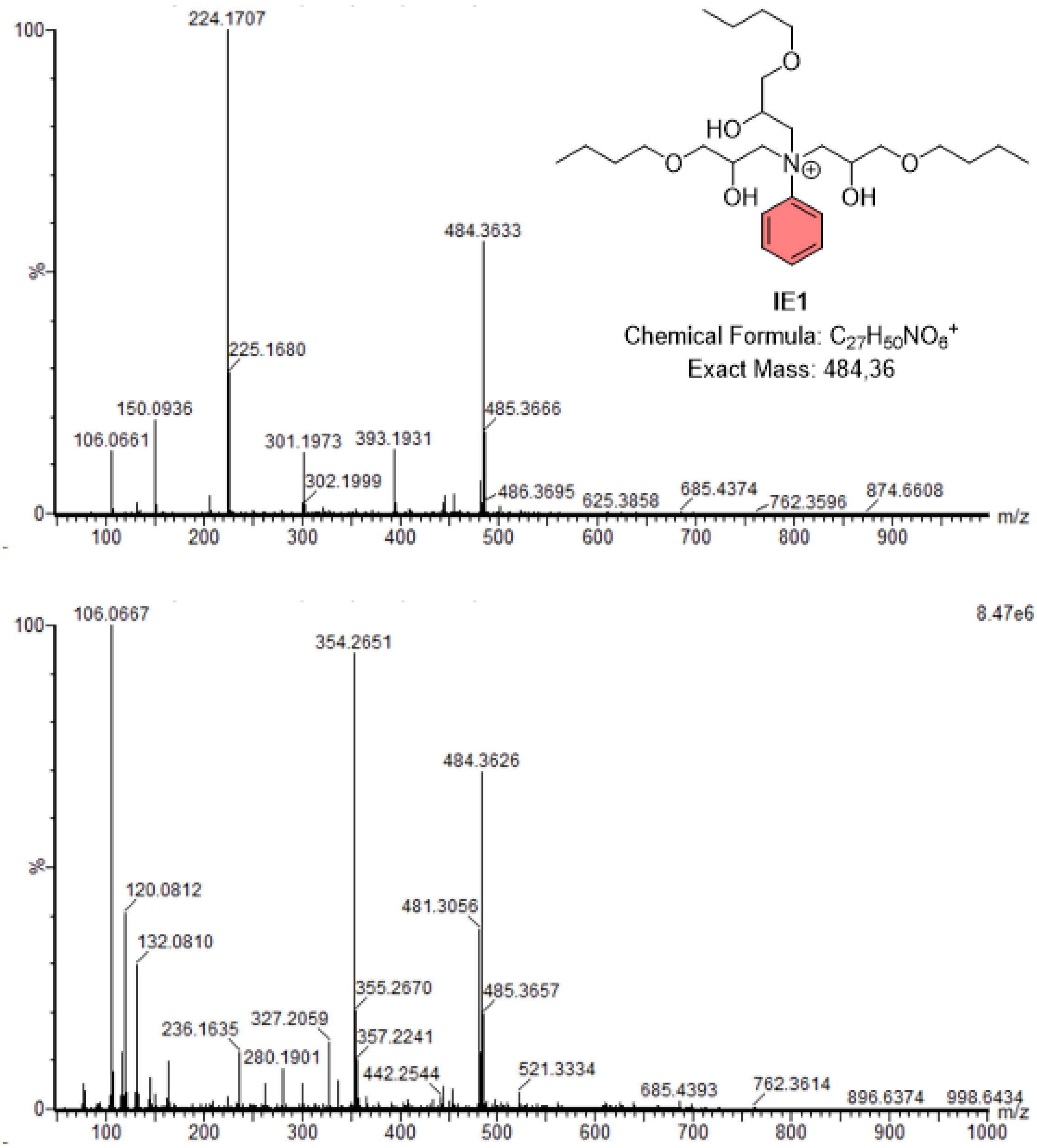

Figure S3. Electrospray ionization mass spectrum (ESI-MS, top) and tandem mass spectrum (MS/MS, bottom) of adduct **IE1** recorded in positive mode. The main peak at *m/z* = 484.36 corresponds to the molecular ion [M^+] of **IE1** ($C_{27}H_{50}NO_6^+$), consistent with a fully substituted quaternary ammonium structure. Notably, no sodium adduct $[M - H + Na]^+$ is observed, supporting the absence of an exchangeable proton on the nitrogen. The MS/MS spectrum displays characteristic fragment ions that confirm the proposed structure.

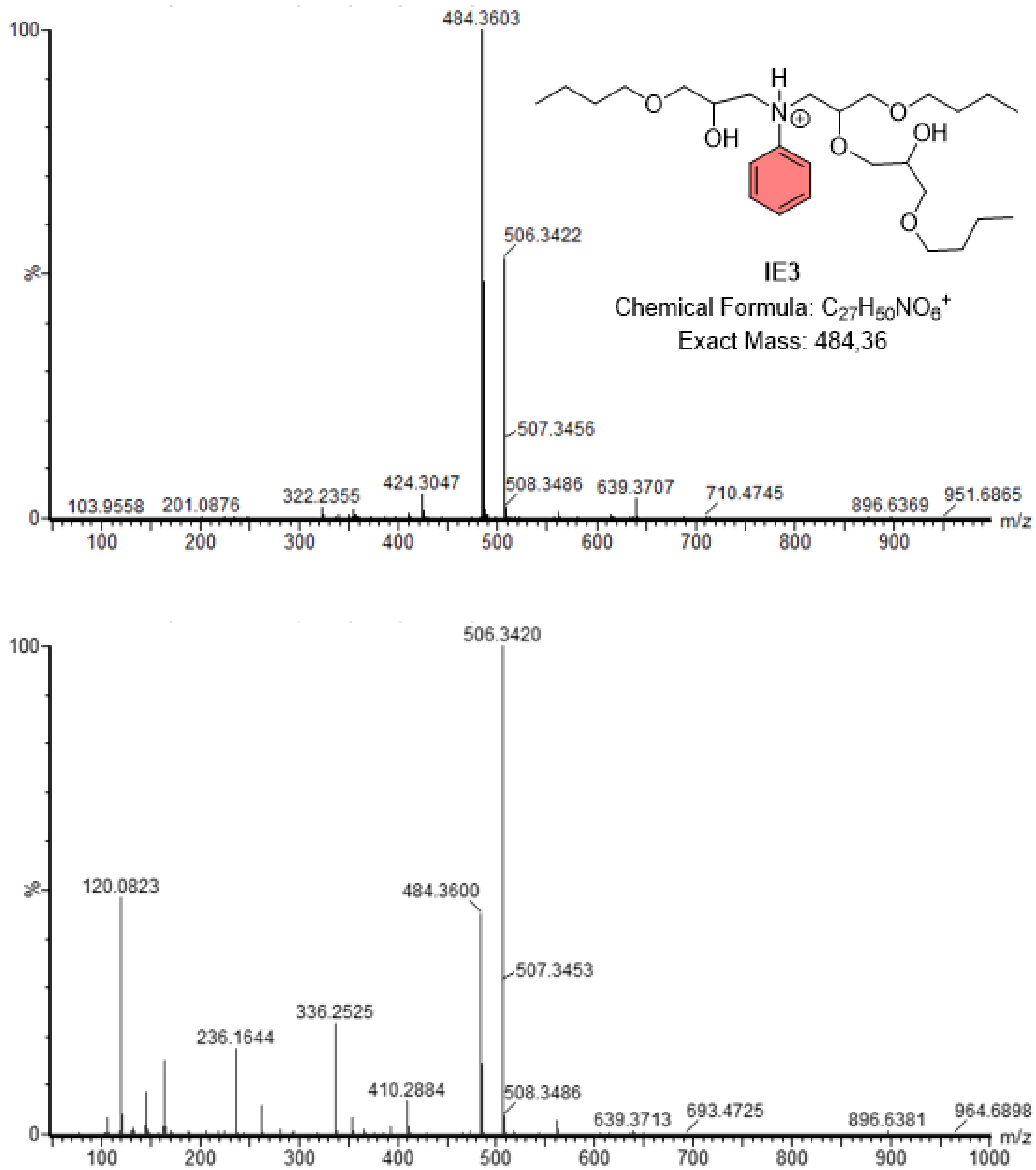

Figure S4. Electrospray ionization mass spectrum (ESI-MS, top) and tandem mass spectrum (MS/MS, bottom) of adduct **IE3** recorded in positive mode. The main peak at *m/z* = 484.36 corresponds to the protonated molecular ion [M^+] of **IE3** ($C_{27}H_{50}NO_6^+$), while the signal at *m/z* = 506.34 is assigned to the sodium adduct $[M - H + Na]^+$. The MS/MS spectrum supports this assignment by showing consistent fragmentation patterns.

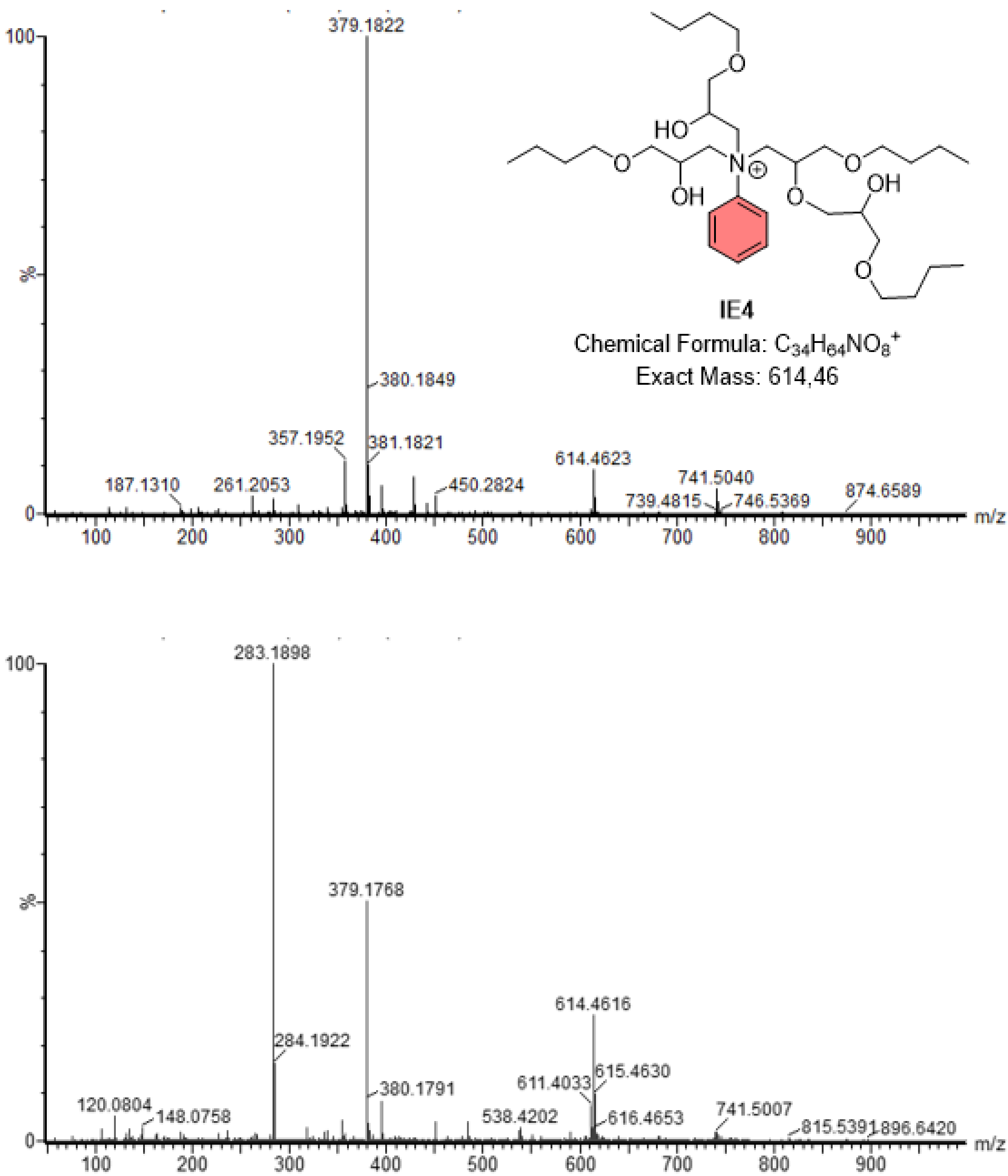

Figure S5. Electrospray ionization mass spectrum (ESI-MS, top) and tandem mass spectrum (MS/MS, bottom) of adduct **IE4** recorded in positive mode. The main peak at *m/z* = 614.46 corresponds to the molecular ion [M^+] of **IE4** ($C_{34}H_{64}NO_8^+$), consistent with a fully quaternized ammonium structure. The absence of a sodium adduct $[M - H + Na]^+$ further supports the lack of exchangeable protons on the nitrogen center. The MS/MS spectrum shows characteristic fragment ions that confirm the structural assignment.

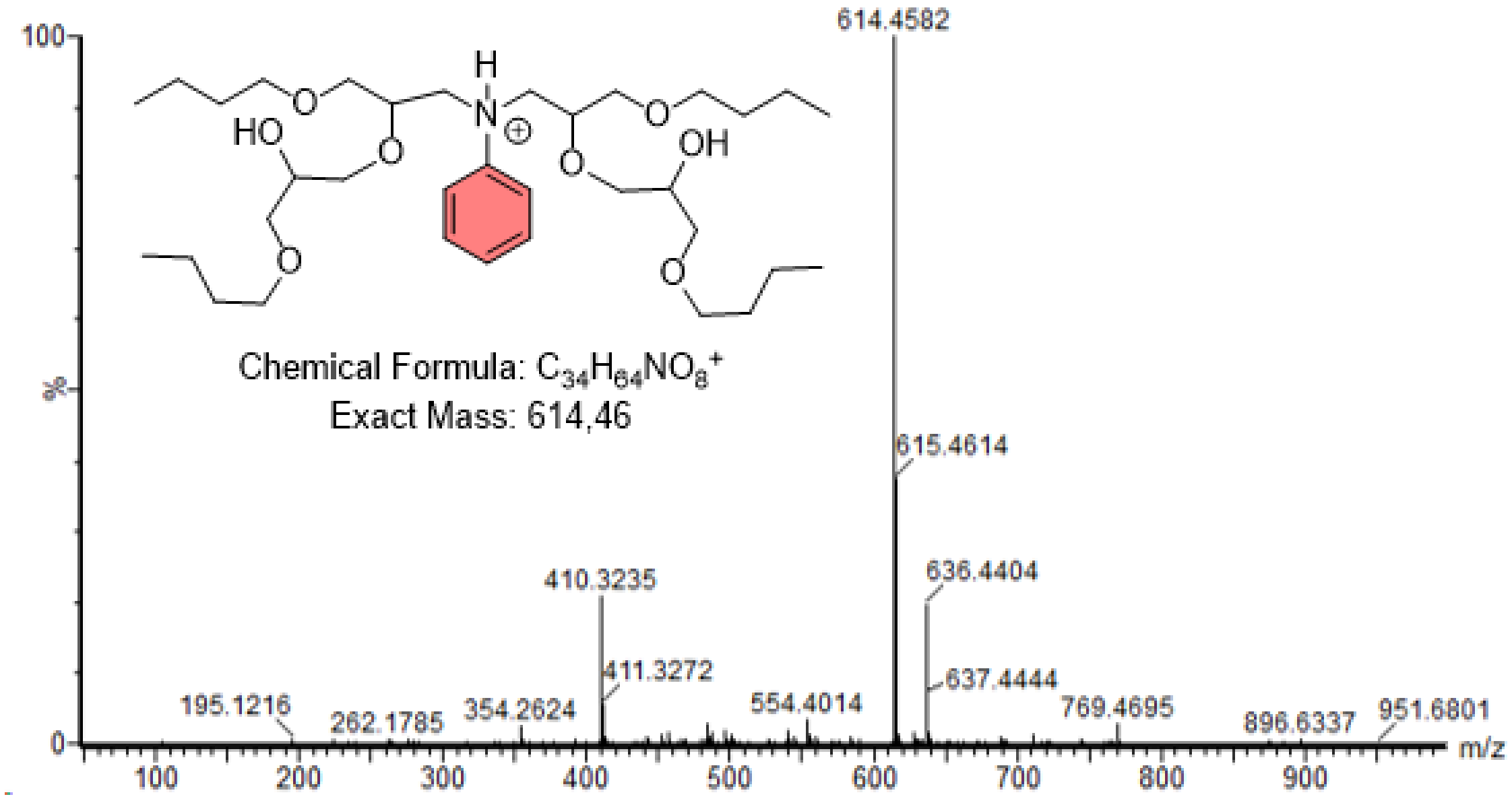

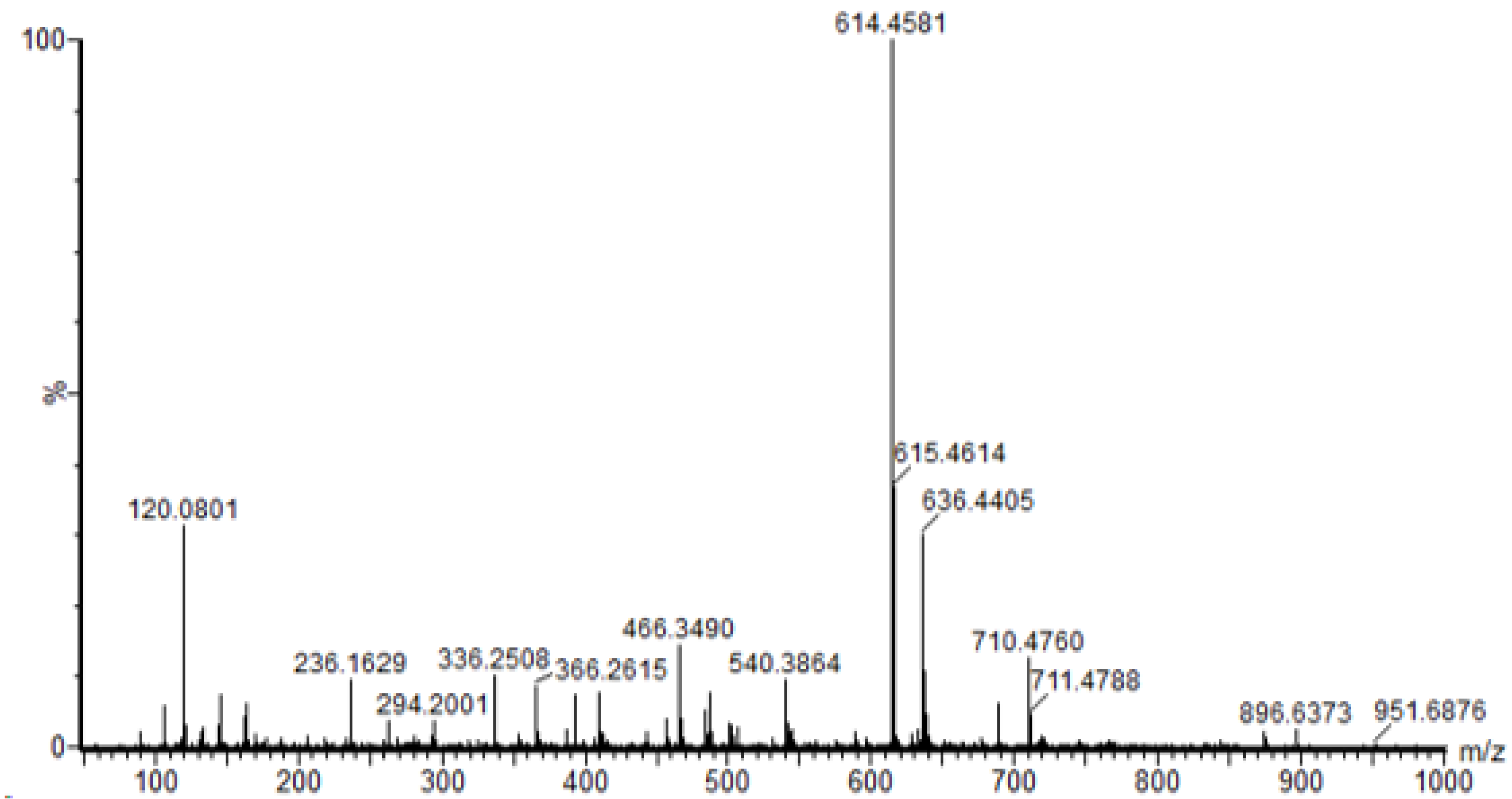

Figure S6. Electrospray ionization mass spectrum (ESI-MS, top) and tandem mass spectrum (MS/MS, bottom) of adduct **IE5** recorded in positive mode. The peak at m/z = 614.46 corresponds to the [M^+] ion of **IE5** ($C_{34}H_{64}NO_8^+$), while a secondary peak at m/z = 636.44 is assigned to the sodium adduct $[M - H + Na]^+$. The presence of this adduct suggests that the ammonium center is not fully quaternized. The MS/MS spectrum confirms the structure through characteristic fragmentation.

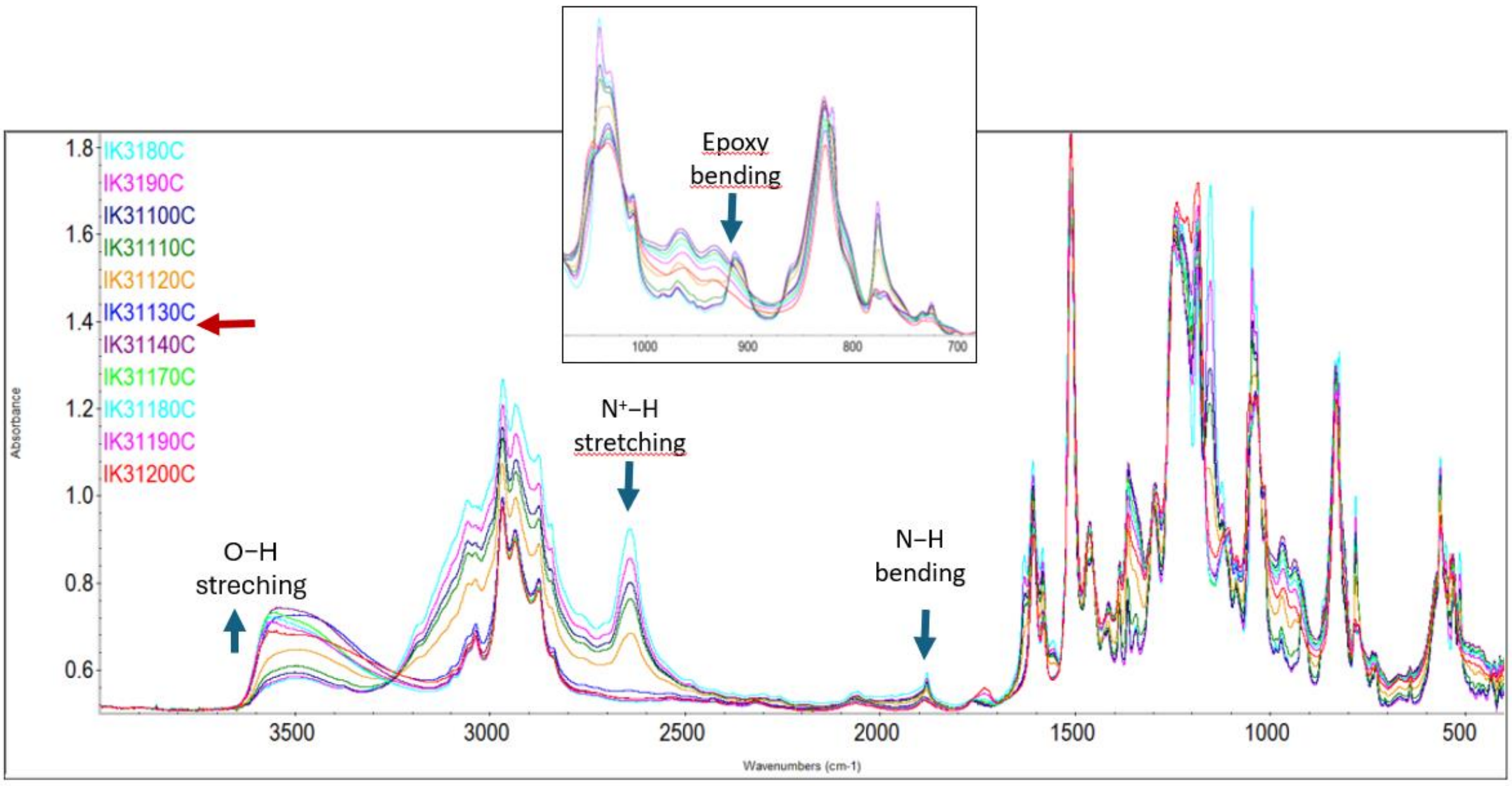

Figure S7. FTIR spectra of the reaction between **DGEBA** and **S1** recorded at different temperatures. A sample of the mixture was spread onto a KBr pellet without any covering. It was then heated to 80 °C for 5 minutes, and the first spectrum was recorded. The temperature was subsequently increased in 10 °C intervals up to 180 °C, holding the sample at each temperature for 5 minutes before recording the corresponding spectrum.

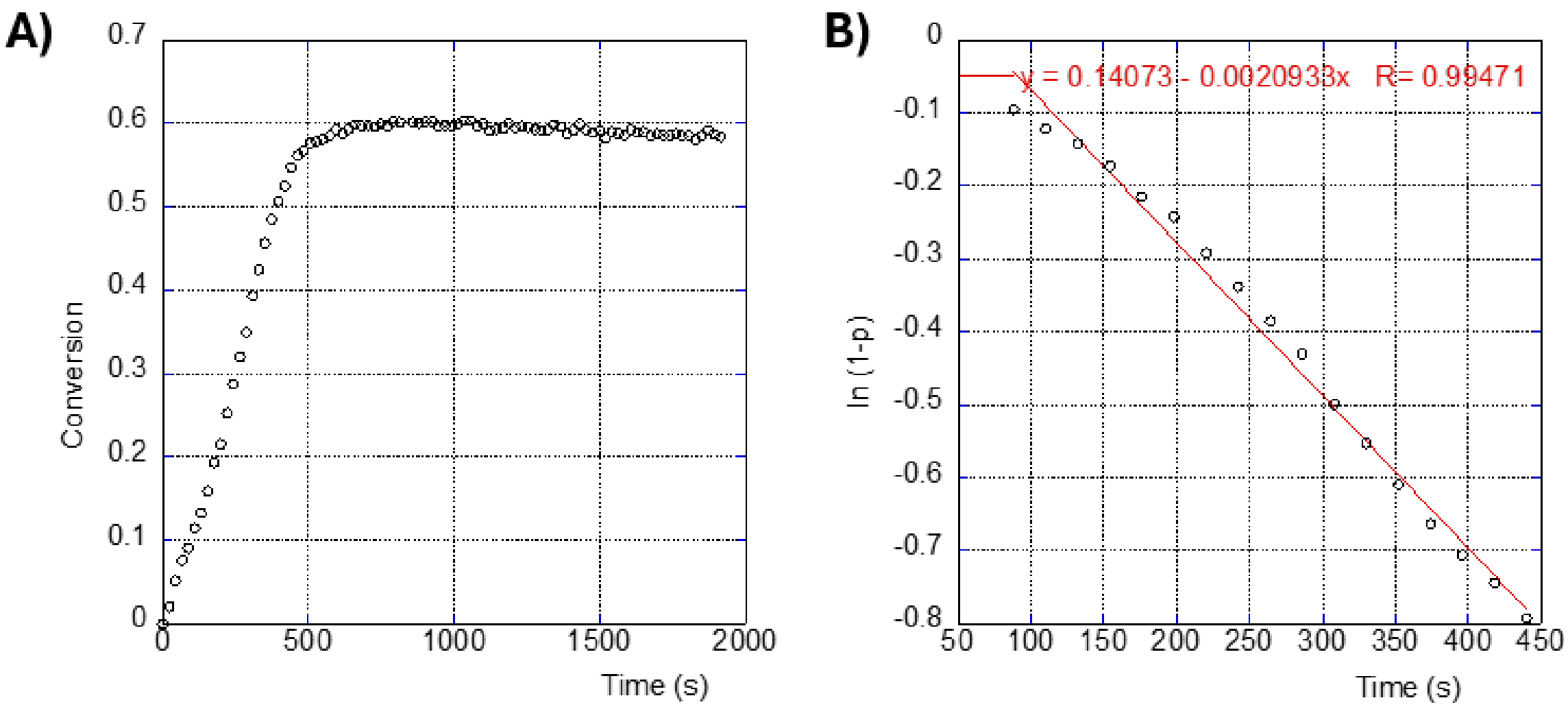

Figure S8. *In-situ* FTIR kinetics of the curing reaction of the DGEBA/**S1** system at 120 °C. **A)** Evolution of the epoxy conversion calculated from the decrease of the 916 cm^{-1} absorption band as a function of time. The conversion reaches approximately 60%, consistent with the expected stoichiometric ratio for the first curing stage. **B)** First-order kinetic plot (ln(1 – α) vs. time) yielding a rate constant $k = 2.1 \times 10^{-3}$ s^{-1} ($R^2 = 0.99$), confirming apparent first-order behavior during the initial step.

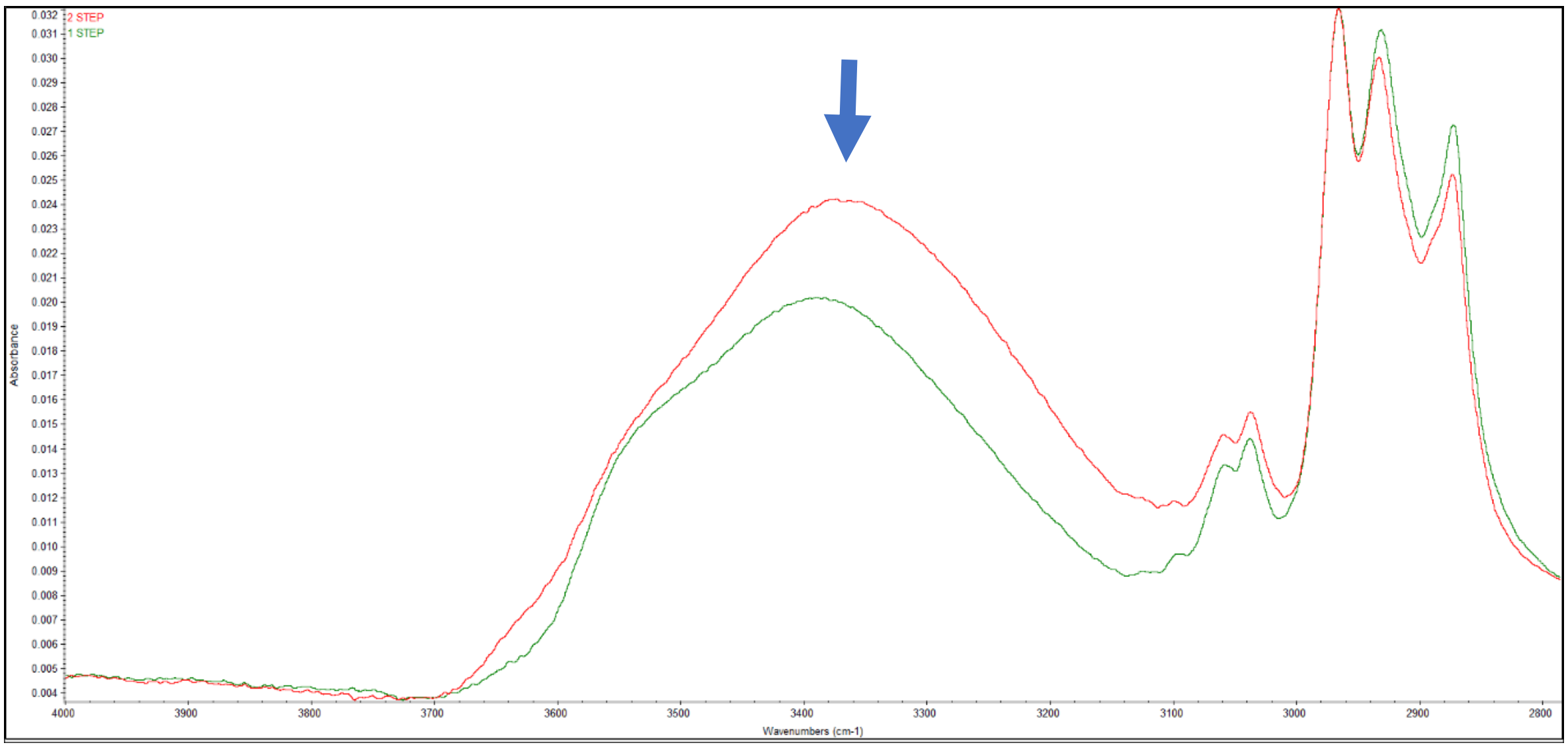

Figure S9. FTIR spectra showing the increase in O–H stretching bands during the transition from **TP1** (green line) to **IEN1** (red line).

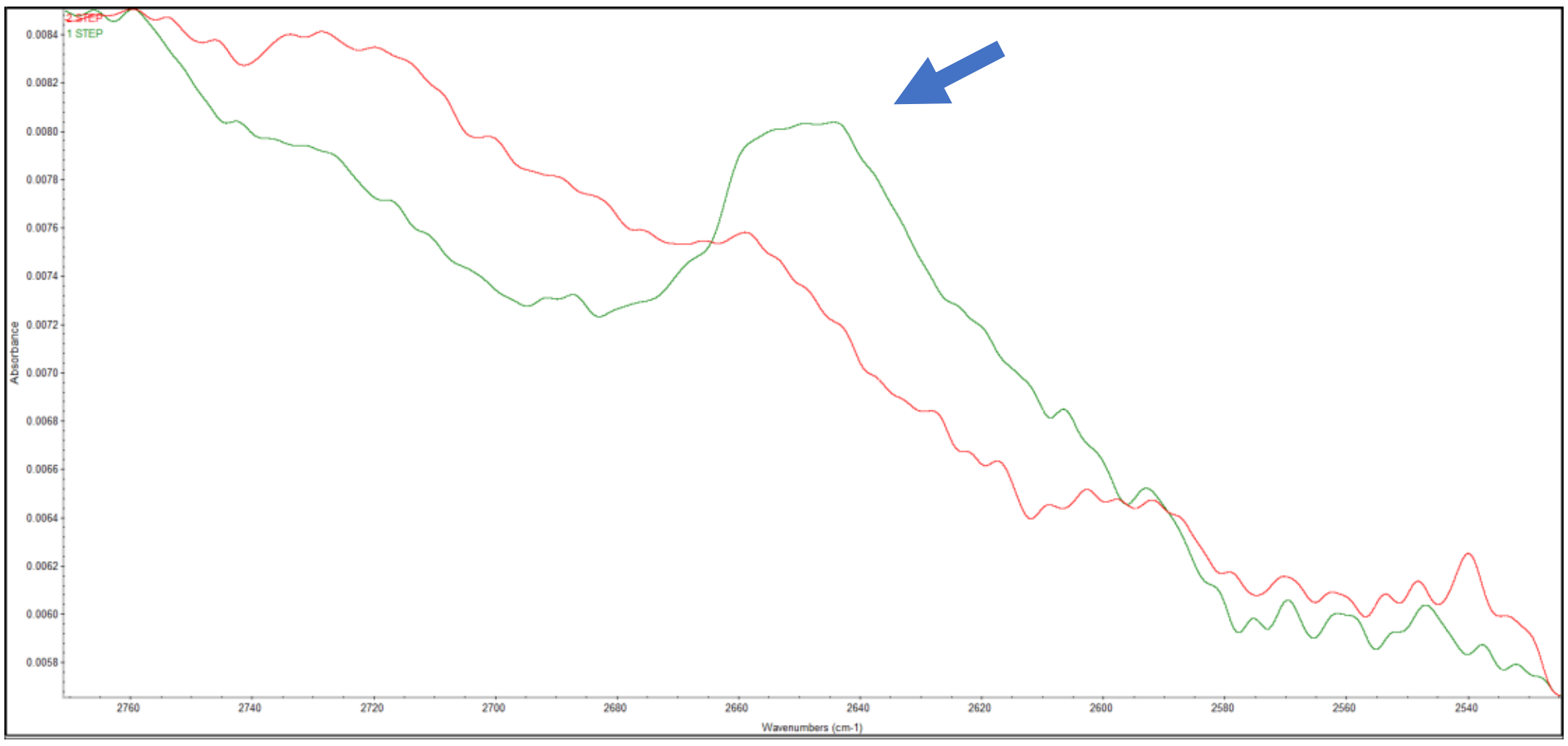

Figure S10. FTIR spectra showing the decrease in N^+–H stretching bands at 2648 cm^{-1}, during the transition from **TP1** (green line) to **IEN1** (red line).

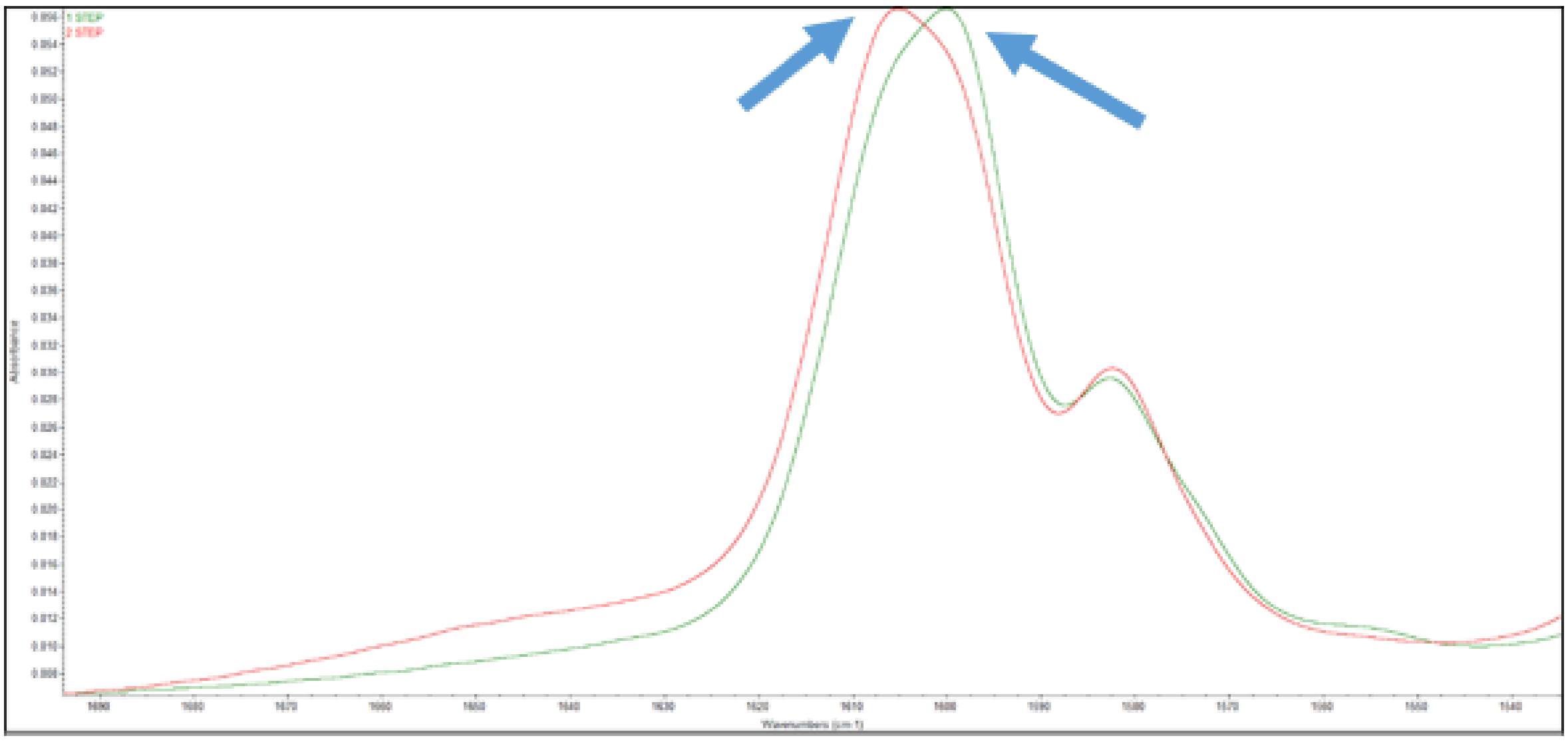

Figure S11. FTIR spectra showing a decrease in the N–H bending band at 1600 cm^{-1} during the transition from **TP1** (green line) to **IEN1** (red line). The band at 1605 cm^{-1} corresponds to C=C stretching of the aromatic ring, while the band at 1600 cm^{-1} is attributed to N–H bending.

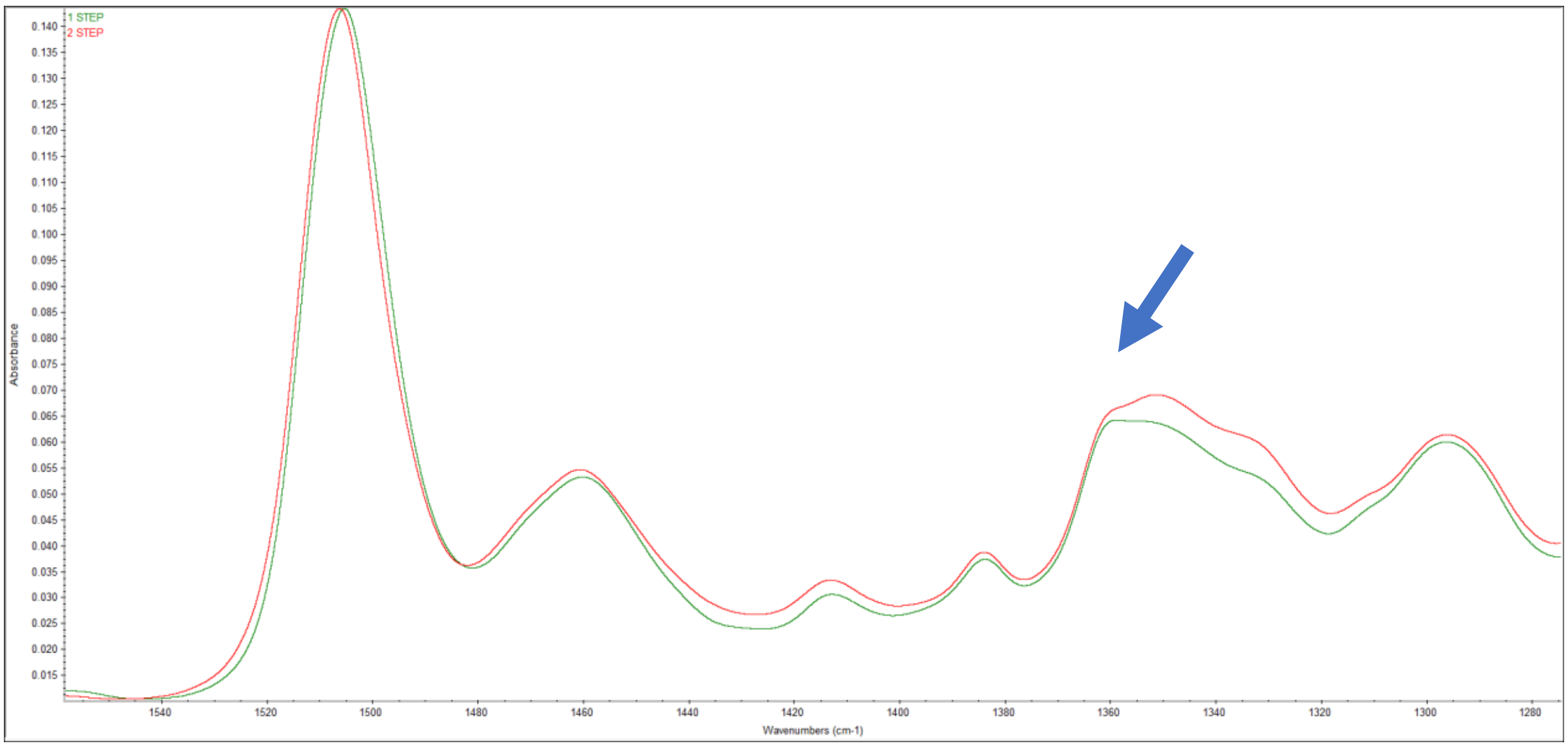

Figure S12. FTIR spectra showing an increase in absorbance in the 1320–1375 cm^{-1} region during the transition from **TP1** (green line) to **IEN1** (red line). This band is associated with C–N stretching vibrations.

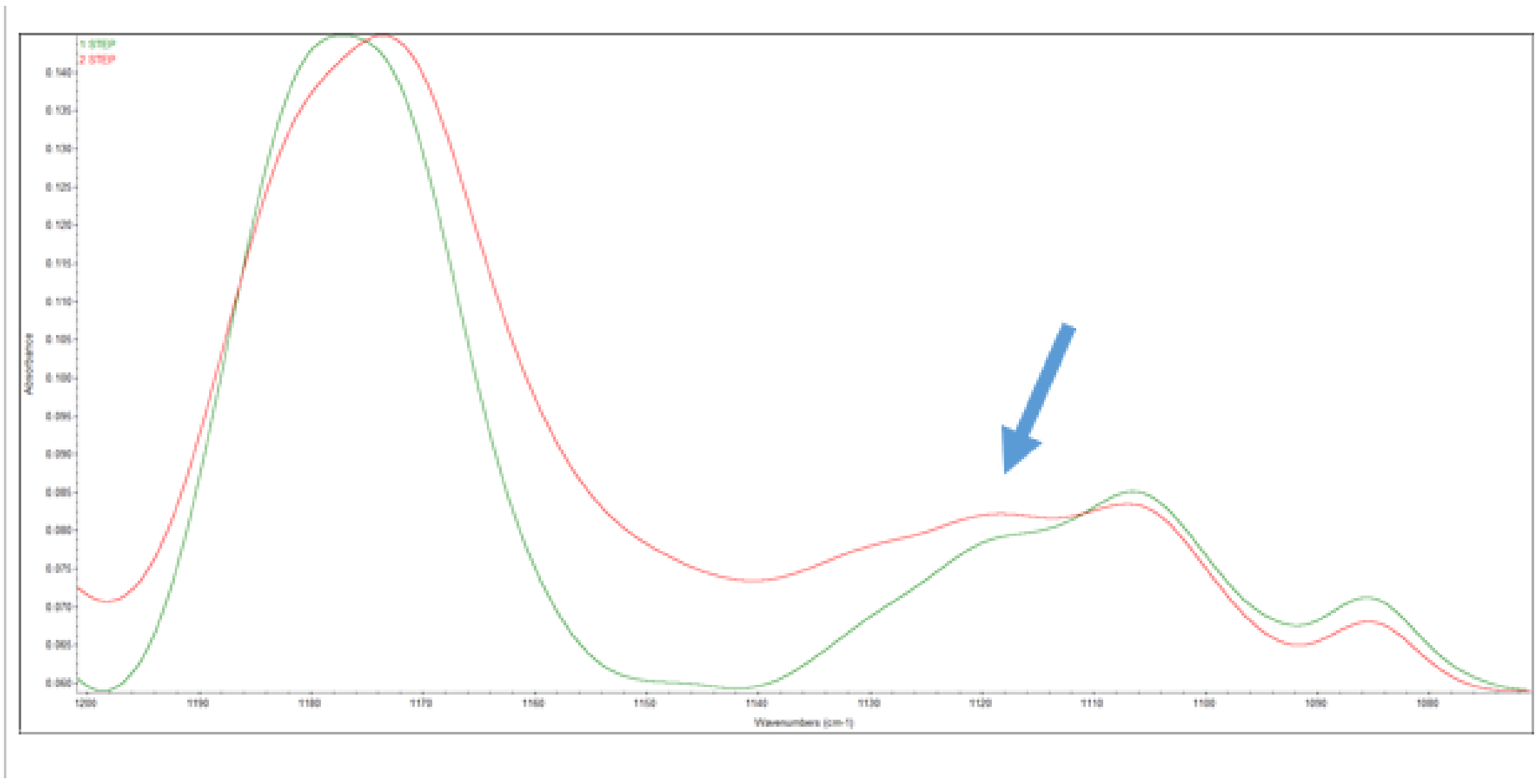

Figure S13. FTIR spectra showing an increase in the C–O stretching band of secondary alcohols in the 1120 cm⁻¹ region during the transition from **TP1** (green line) to **IEN1** (red line).

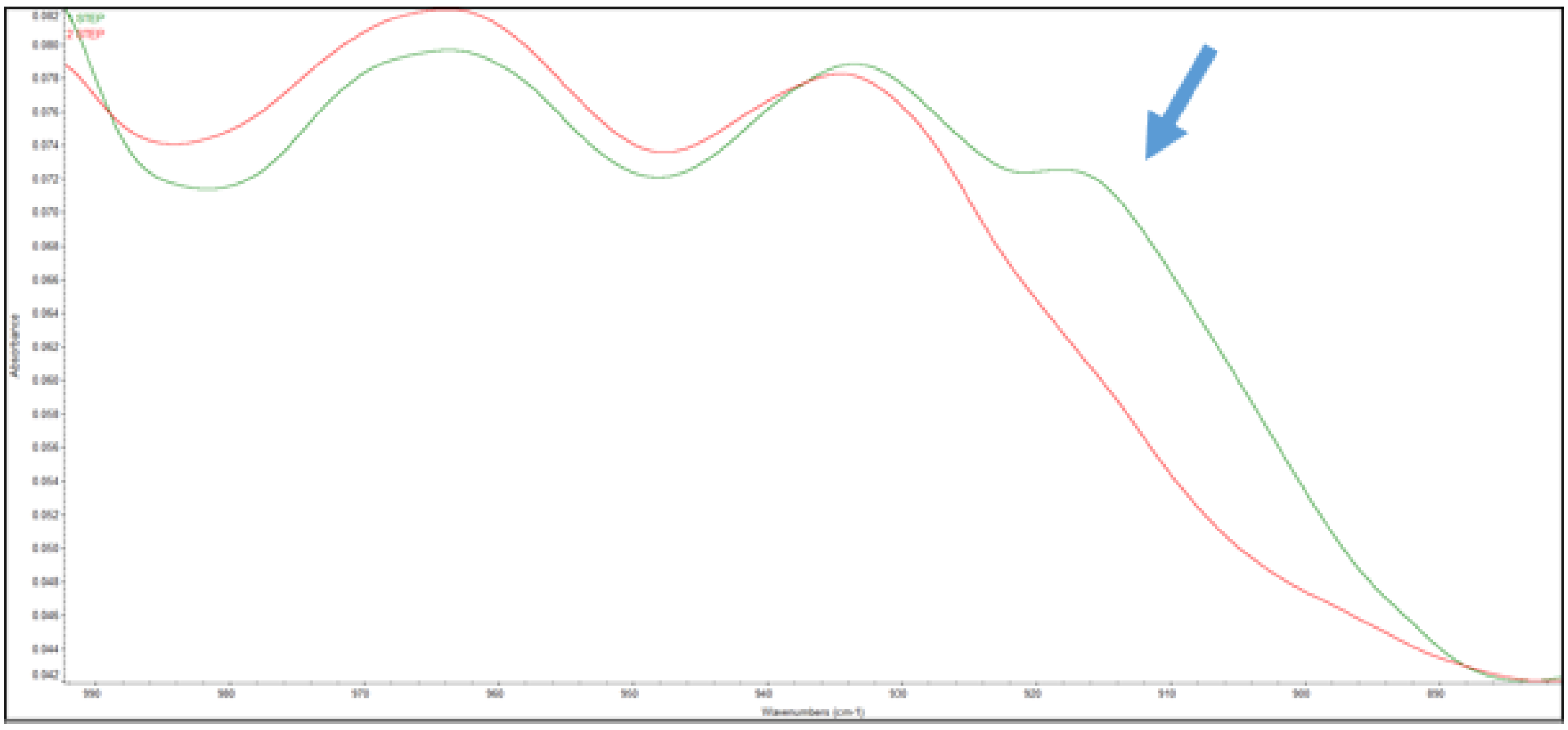

Figure S14. FTIR spectra showing a decrease in the characteristic epoxy ring bending band at 916 cm⁻¹ during the transition from **TP1** (green line) to **IEN1** (red line).

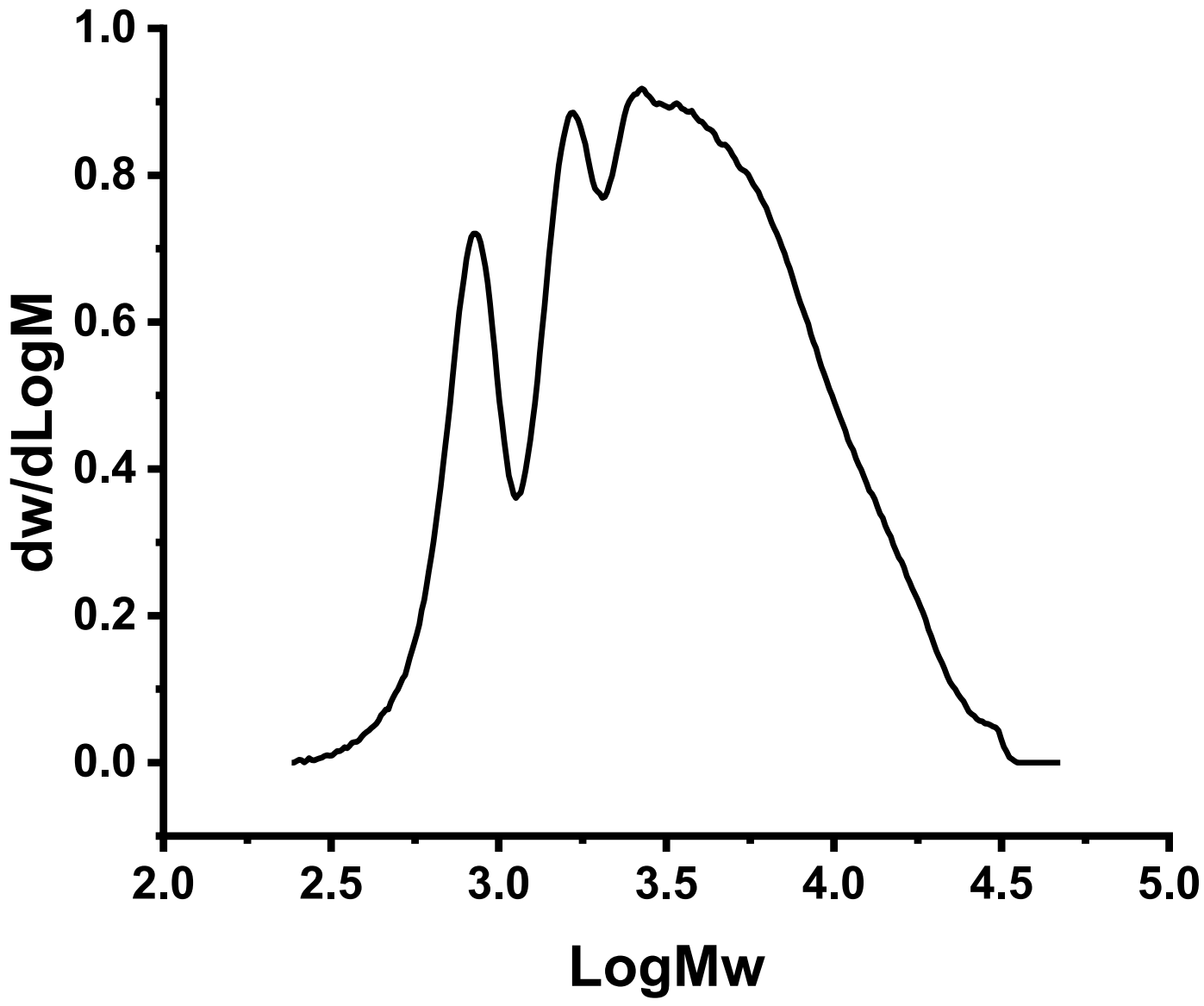

Figure S15. Molecular weight distribution of **TP1**, estimated by size exclusion chromatography (SEC) in chloroform.

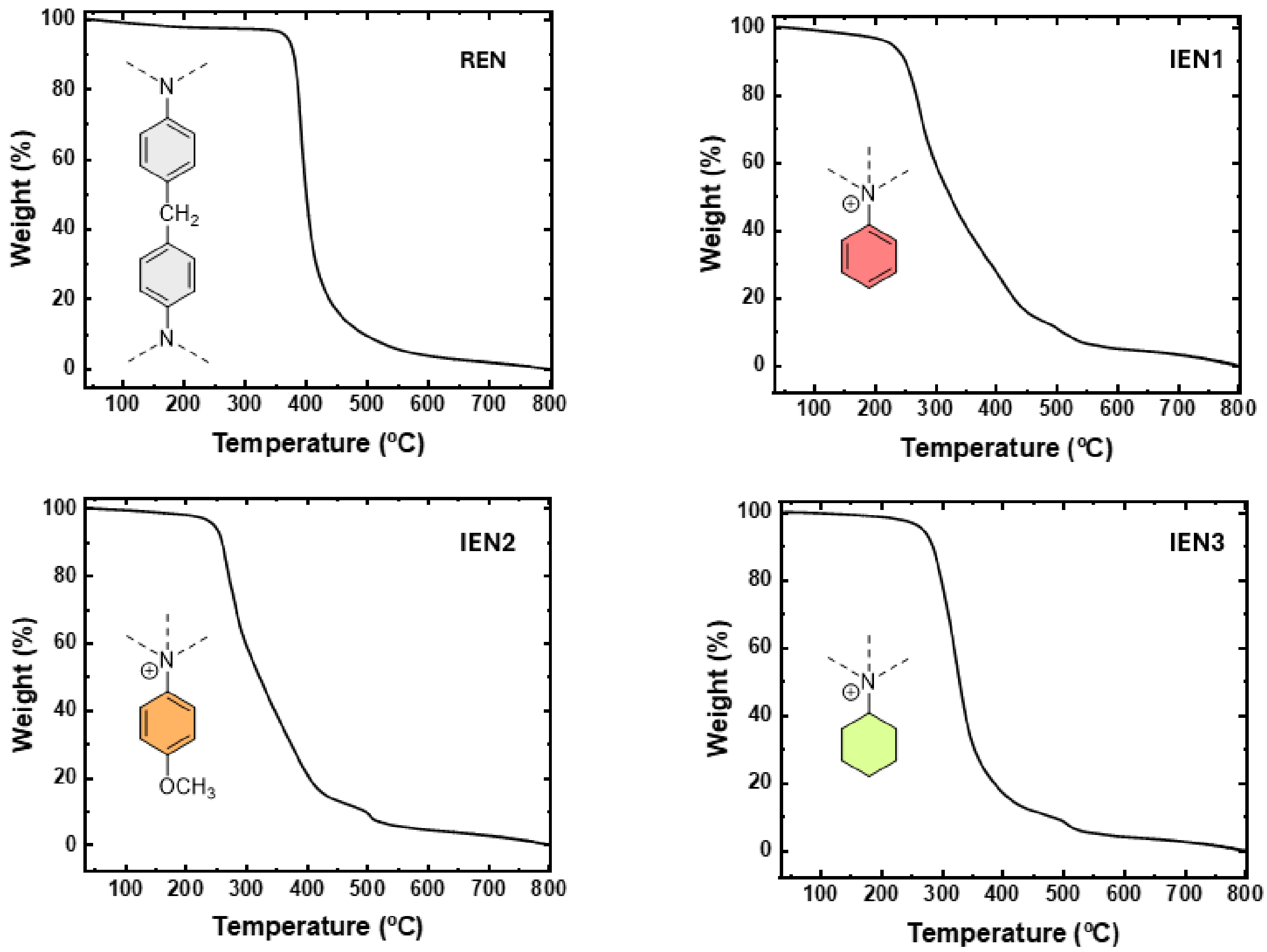

Figure S16. TGA plots of **REN**, **IEN1**, **IEN2**, and **IEN3**.

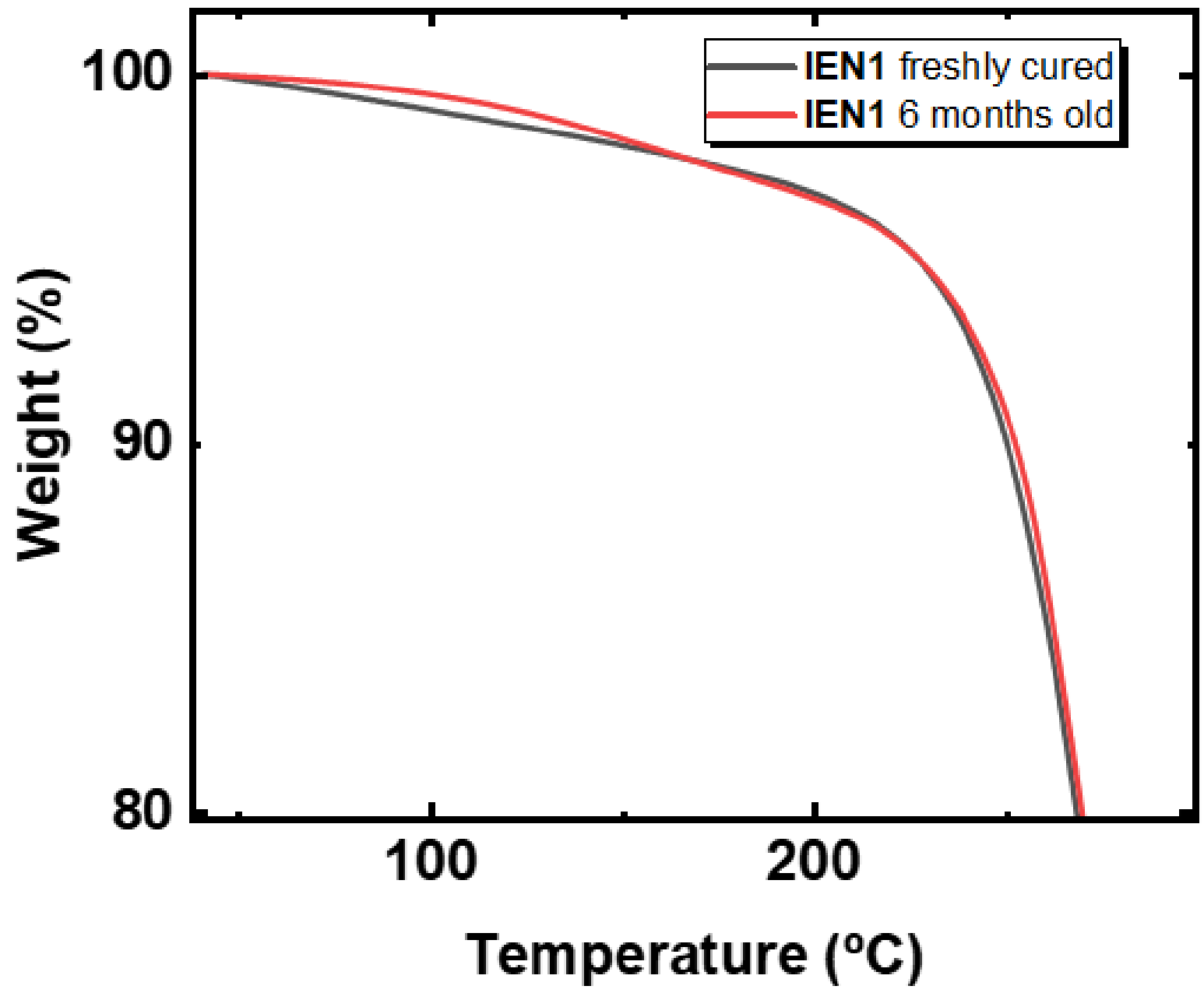

Figure S17. Low-temperature region of the TGA curves of **IEN1** for freshly cured (black) and six-month-aged (red) samples stored under ambient laboratory conditions (20–26 °C, 70–80% RH).

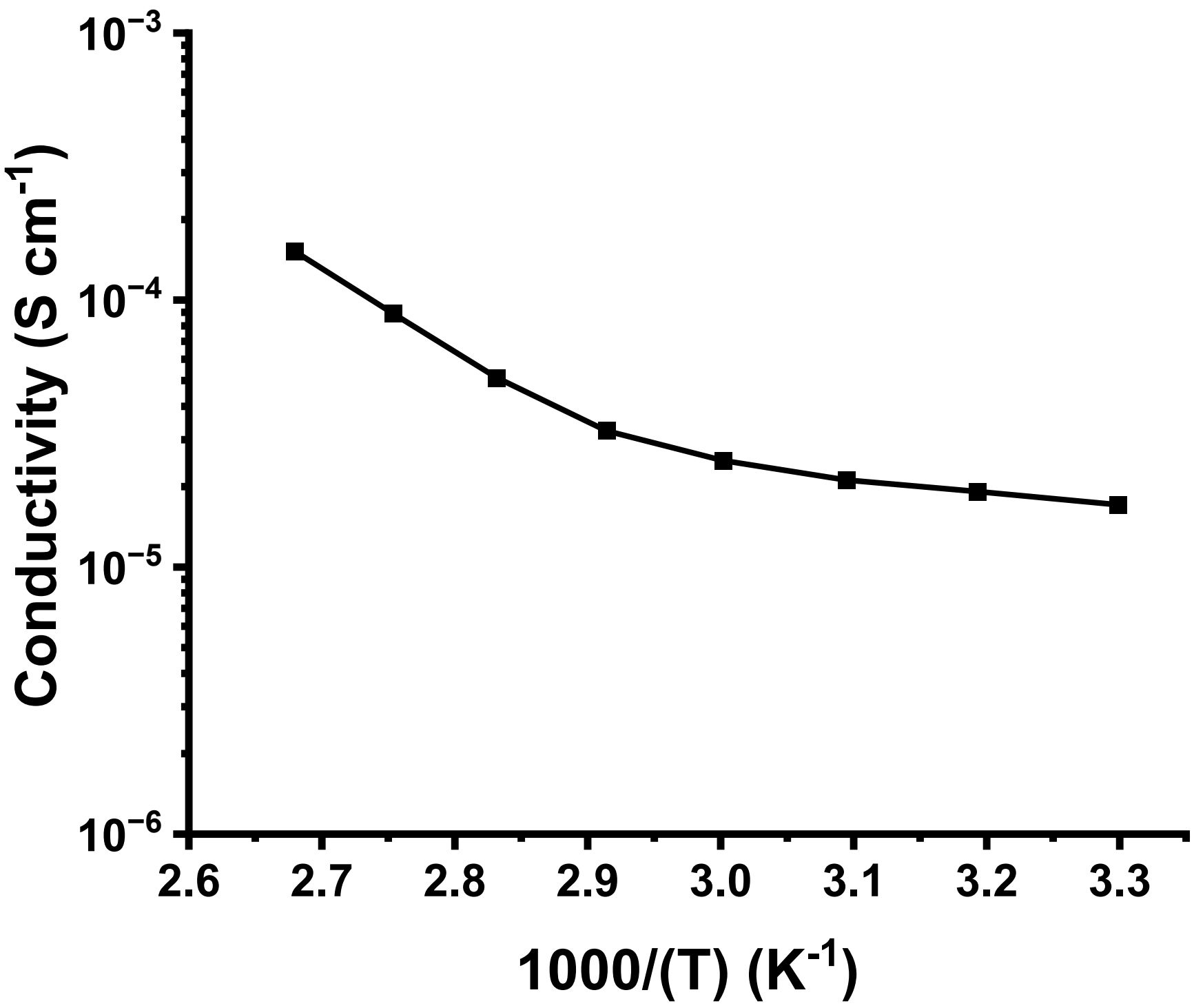

Figure S18. Ionic conductivity as a function of temperature of **IEN1** measured by electrochemical impedance spectroscopy (EIS).

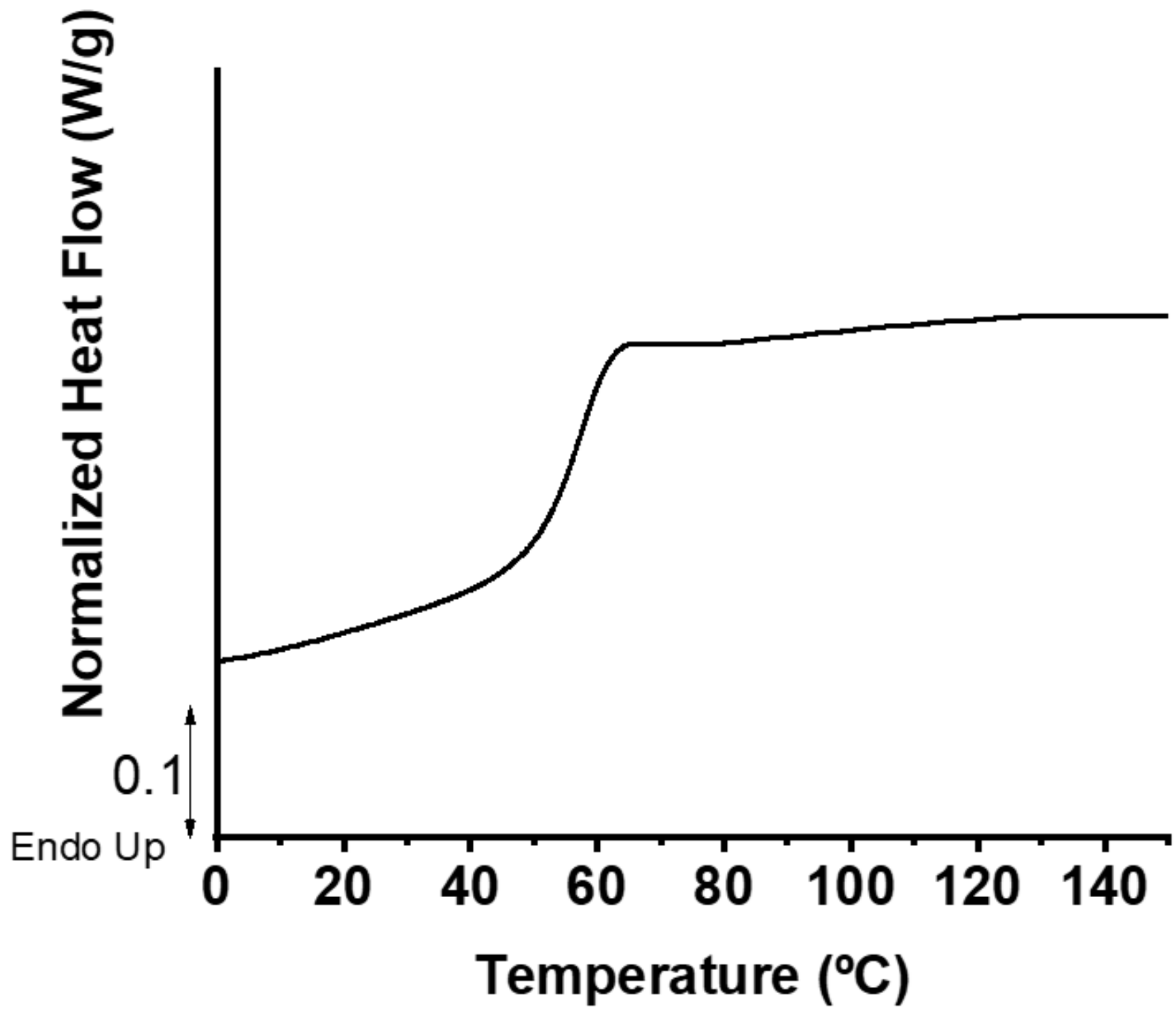

Figure S19. DSC trace (5 ºC min^{-1}) of the intermediate **TP1**, showing a glass transition temperature (T_g) of 56 ºC.

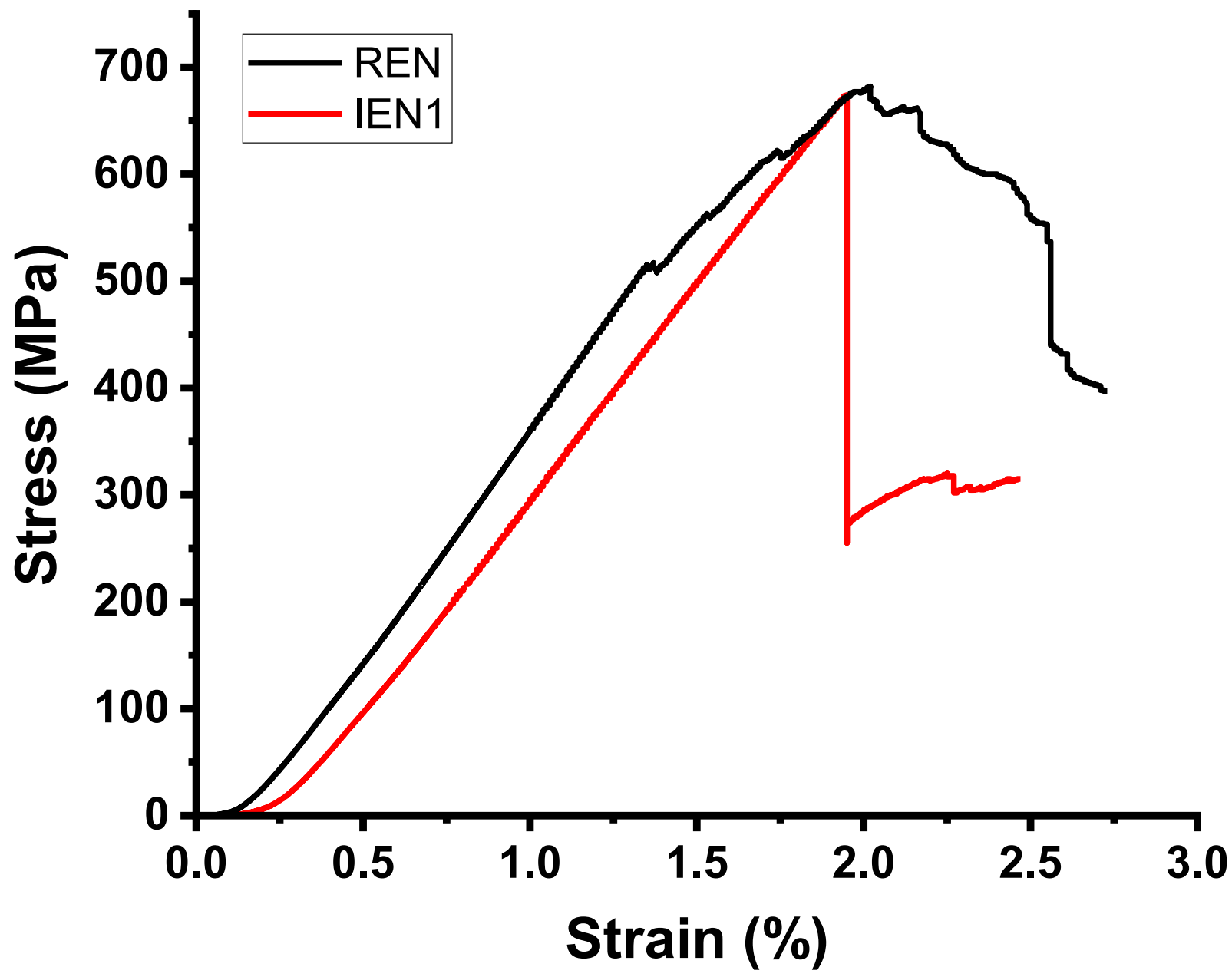

Figure S20. Representative stress–strain curves from three-point bending tests of 10-ply carbon fiber-reinforced composites based on **REN** (black solid line) and **IEN1** (red solid line). All composites exhibit nearly identical mechanical behavior, with comparable flexural strength and strain at break.

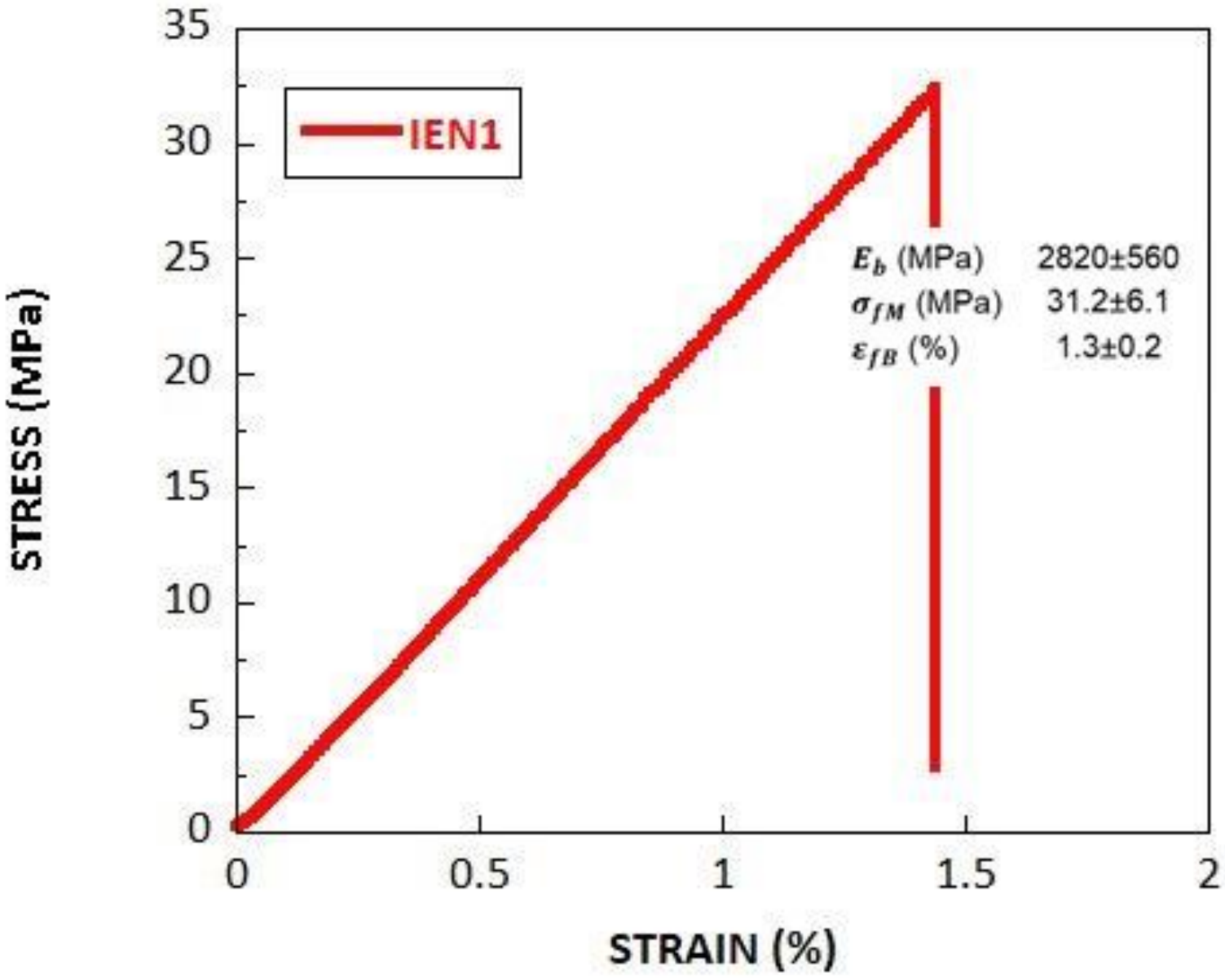

Figure S21. Representative three-point bending stress–strain curve of the cured ionic epoxy network **IEN1**. The material exhibits a flexural modulus (E_b = 2.8 ± 0.6 GPa), flexural strength (σ_{fM} = 31.2 ± 6.1 MPa), and strain at break (ε_{fB} = 1.3 ± 0.2 %).
